# Combining NMR Spectroscopy and Chemometrics to Monitor Structural Features of Crude Hep-arin

**DOI:** 10.3390/molecules22071146

**Published:** 2017-07-08

**Authors:** Lucio Mauri, Maria Marinozzi, Giulia Mazzini, Richard E. Kolinski, Michael Karfunkle, David A. Keire, Marco Guerrini

**Affiliations:** 1 Institute for Chemical and Biochemical Research G. Ronzoni, via G. Colombo 81, 20133 Milan, Italy; mauri@ronzoni.it (L.M.); maria.marinozzi90@gmail.com (M.M.); giulia.mazzini@gmail.com (G.M.); 2 Division of Pharmaceutical Analysis, Office of Testing and Research, Center for Drug Evaluation and Research, U.S. Food and Drug Administration, 645 S. Newstead Ave., St. Louis, MO 63110, USA; Richard.kolinski@fda.hhs.gov (R.E.K.); michael.karfunkle@fda.hhs.gov (M.K.); David.Keire@fda.hhs.gov (D.A.K.)

**Keywords:** heparin, crude heparin, NMR, quantitative NMR, PCA, chemometric, HSQC

## Abstract

Because of the complexity and global nature of the heparin supply chain, the control of heparin quality during manufacturing steps is essential to ensure the safety of the final active pharmaceutical ingredient (API). For this reason, there is a need to develop consistent analytical methods able to assess the quality of heparin early in production (i.e., as the crude heparin before it is purified to API under cGMP conditions). Although a number of analytical techniques have been applied to characterize heparin APIs, few of them have been applied for crude heparin structure and composition analyses. Here, to address this issue, NMR spectroscopy and chemometrics were applied to characterize 88 crude heparin samples. The samples were also analyzed by strong anion exchange HPLC (SAX-HPLC) as an orthogonal check of the purity levels of the crudes analyzed by NMR. The HPLC data showed that the chemometric analysis of the NMR data differentiated the samples based on their purity. These orthogonal approaches differentiated samples according their glycosaminoglycan (GAG) composition and their mono and disaccharide composition and structure for each GAG family (e.g., heparin/heparan, dermatan sulfate, and chondroitin sulfate A). Moreover, quantitative HSQC and multivariate analysis (PCA) were used to distinguish between crude heparin of different animal and tissue sources.

## 1. Introduction

In spite of its 100 year history, heparin and its lower molecular weight versions remain the most used anticoagulant and antithrombotic drug [1]. Heparin is a sulfated polysaccharide composed of alternating disaccharide sequences of uronic acid (either d-glucuronic or l-iduronic acid) and glucosamine. The glucosamine residue can be *N*-acetylated or *N*-sulfated in position 2, while both uronic acid and glucosamine can be sulfated in position 2 and 6, respectively. More rarely, the glucosamine residue can be sulfated in position 3, and found in the pentasaccharide sequence GlcNS/Ac-GlcA-GlcNS,3S,6S/OH-IdoA2S-GlcNS,6S, corresponding to the heparin binding site for antithrombin (AT). The degree of sulfation and distribution within the heparin chains depends on the animal or organ source; however, the detailed monosaccharide sequence is still largely undisclosed [2].

Until the late 1980s and early 1990s, when cases of bovine spongiform encephalopathy (BSE) were reported in United Kingdom and other countries, bovine heparin products were widely used together with those extracted from porcine intestine. As a result of BSE, bovine heparin was withdrawn from both the U.S. and European markets and entirely replaced by porcine mucosa heparin, limiting bovine heparin use to South America and some Islamic countries. The removal of bovine heparin from the market increased the market demand for porcine heparin. As a result, at present more than 50% of worldwide heparin production originates from China [3]. In addition, a shortage in the heparin supply (due also to a pig disease outbreak) was presumably the reason for the heparin contamination with the semisynthetic over-sulfated chondroitin sulfate (OSCS), an inexpensive adulterant with anticoagulant activity, which occurred in late 2007 in the US and Europe [4]. This so called heparin crisis, in addition to causing many deaths and severe side effects, demonstrated the vulnerability of the global heparin supply chain. Fortunately, the subsequent introduction of new tests (e.g., NMR and HPLC tests) to the heparin monographs of several pharmacopoeias around the world increased the degree of quality control of this drug, decreasing the risk of product adulteration [3]. However, the complexity of the heparin supply chain provides other opportunities for intentional adulteration, such as contamination with foreign substances or the addition of non-porcine sources of crude heparin (i.e., bovine or ovine heparin), which might not be detected by the current monograph test methods.

Heparin is produced from tissues of food animals, and for medical use the drug has been primarily obtained from pig or beef intestine, and can be sourced from sheep as well. Typically, the pig intestine is collected and processed in slaughterhouses approved by regulatory authorities and then transported to crude heparin processing facilities. In these facilities, the intestinal mucosa is removed and the dissociated mucosa is treated with proteolytic enzymes under alkaline conditions. After multiple steps, the highly anionic glycosaminoglycans (GAGs) can be adsorbed onto an anion exchange resin, which is washed. Then, the partially purified heparin is eluted, filtered, precipitated, and vacuum dried to form a substance known as crude heparin, where the glycosaminoglycan (GAG) fraction is concentrated. Crude heparin is then further purified at the level of active pharmaceutical ingredient (API) to remove impurities such as dermatan and chondroitins, nucleic acids, residual proteins, metal ions, and unwanted counterions. In addition, the purification involves oxidation (KMnO_4_ or H_2_O_2_) and alkaline treatments to remove color and inactivate endotoxins and viruses that might be present in the product [5,6].

Whereas the purification of crude heparin to heparin sodium API is always conducted under cGMP conditions, only a few manufacturers can establish traceability back to the living animals. Many companies buy crude heparins from third parties, which have collected mucosa at multiple small slaughterhouses. These multiple sites increase the risk of un-intended or intentional contamination. Although it is impossible to eliminate all possible risks, reinforcing of appropriate requirements, controls, and best practices at the level of crude heparin as a key intermediate might help ensure the quality of the final pharmaceutical grade product.

A first step to define crude heparin as an intermediate in the production process of the API requires the specification of a normal range of composition found in heparin currently on the market. Different intermediates may exist and be qualified for use in the manufacture of heparin, such as resin bound heparin (early intermediates), a brown powder containing a large amount of RNA/DNA and galactosaminoglycans (mainly dermatan sulfate (DS) and chondroitin sulfate A (CSA)), or partly purified crude heparin, a light yellow powder containing only a small amount of dermatan sulfate [7]. Although different techniques have been used to identify and quantify OSCS in crude heparin, to our knowledge, these studies did not fully characterize the average composition of crudes produced in the global marketplace [8,9].

In the present study, 88 crude heparins collected from 2010 to 2015, representing 13 different manufacturers, have been characterized by NMR spectroscopy and strong anion exchange HPLC (SAX-HPLC). Particularly, a multivariate analysis (principal component analysis (PCA)) of one-dimensional (1D) proton NMR spectra was used to differentiate the crudes on the basis of their structural features and animal/organ of origin. Moreover, the quantitative composition of crude samples was performed using an adapted version of the HSQC method recently described in [10].

## 2. Results

### 2.1. Protocol for Sample Preparation

Crude heparin samples are often not completely soluble in water. Therefore, to avoid the presence of insoluble material in the NMR tube, a protocol of sample separation by centrifugation was developed. Three parameters of the separation protocol were evaluated for their effect on the supernatant content, including: the amount of insoluble material present, crude concentration, and the method of solution equilibration (with or without stirring). Each of the eight combinations of the parameters was tested. Moreover, three separations were performed for a single parameter set to evaluate the precision of the data, giving a total of ten separations. For each separation, two parameters were measured after lyophilization of the supernatant or the pellet: the total weight loss and the amount of precipitate, both expressed as a percentage of the original weight (Table 1).

With the exclusion of one result, the loss of water due to lyophilization was around 10%, regardless of sample concentration. While the stirring procedure during solution equilibration did not affect the yield on a percentage basis, the amount of precipitate was related to the sample concentration, showing that the precipitate still contains soluble material. To verify that the procedure does not result in the selective separation of glycosaminoglycan species, a precipitate obtained at a higher concentration (80 mg/mL) was re-suspended in 1 mL of D_2_O, centrifuged, and the spectrum of the supernatant compared with that obtained after the first solubilization. While no differences in the glycosamonoglycan pattern were found, higher non-GAG signals in the re-suspended precipitate were observed (Figure S1). Based on these results, to collect enough soluble material for the NMR characterization, an aliquot of 60 mg of crude heparin was determined to be sufficient for analysis in 1.5 mL of D_2_O. The average weights of the 88 crude samples are shown in Table S1.

The proton chemical shifts of heparin are affected both by the pH and concentration of the solution [11,12]. Crude heparin does not contain only heparin, but also other GAGs (heparan, dermatan, and chondroitin sulfate), as well as non-GAG material, mainly DNA and RNA, some proteinaceous material, and a variable amount of water and salts. For these reasons, the proton spectra of crude heparins in D_2_O were not fully reproducible, and a shift of signals, particularly those of anomeric glucosamine and iduronic acid residues, was often observed (Figure S2). The maximum shift of both peaks observed was about 10–12 Hz (0.015–0.020 ppm), presumably due to the different concentration of GAG components in the samples, which affect the pH of the solution. These spectra cannot be easily compared, particularly when they are analysed by multivariate analysis (e.g., PCA). To remove the effect of pH variability, the samples were solubilized in 0.15 M of phosphate buffer at pH 7.1, containing 3 mM of perdeuterated EDTA. The complexation of bivalent or paramagnetic ions induced by the addition of perdeuterated EDTA provides narrower lines and improved resolution. A comparison of crude proton spectra recorded in water and the buffer solution is shown in Figure S2.

### 2.2. One-Dimensional (1D) Spectra Library and PCA

**Figure 1:**
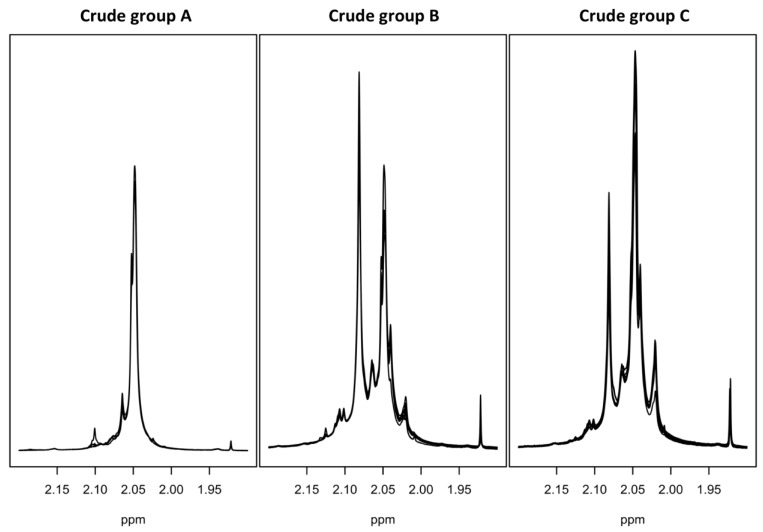
^1^H-NMR spectra. Acetyl region of proton spectra of samples of group A, group B, and group C (see Figure 2) registered at 600 MHz, showing methyl signals of Dermatan (2.08 ppm), Heparin (2.05 ppm), and Chondoritin (2.02 ppm) components.

The proton spectra of eighty-eight crude heparin samples were registered by the NMR analysis protocol indicated in the experimental section. The spectra show very high variability of composition, both in terms of non-GAGs components (broad signals at both aromatic and aliphatic regions due to DNA/RNA and other impurities, Figure S1) and galactosaminoglycans components, mainly dermatan and chondroitin sulfate (Figure 1).

Due to this variability and the difficulties in establishing quantitative ratios among diagnostic signals, the spectral complexity was reduced to the basic elements which best define the crucial differences between samples by employing chemometric techniques.

One of the most common tools used to explore a complex dataset is principal component analysis (PCA). Typically, this tool takes a large number of correlated variables and transforms the data into a smaller number of uncorrelated variables (principal components), while extracting the maximal amount of variation, thus making it easier to analyse the data and make predictions [13].

**Figure 2:**
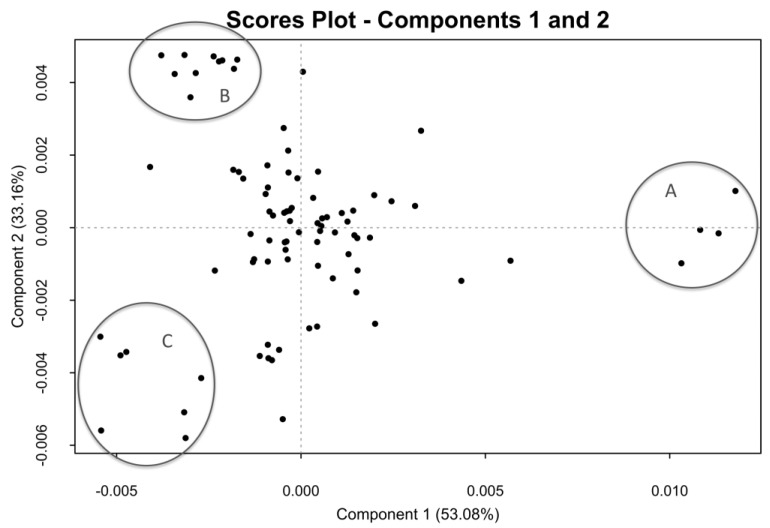
Score plot of the first two components generated by principal component analysis (PCA) of the GAGs signals region of the ^1^H-NMR spectrum. Most of the samples are centered in the PCA, while there are 21 more peripheral samples: highlighted as A, B, and C.

The score plot of the first two components, generated by the PCA of 88 ^1^H-NMR spectra of the crude samples, is shown in Figure 2. The graph, displaying the variance accounted for by the principal components, shows that only two components are necessary to account for more than 80% of the total variance, indicating that the differences among the samples are mainly distributed along the components **1** and **2**.

**Figure 3:**
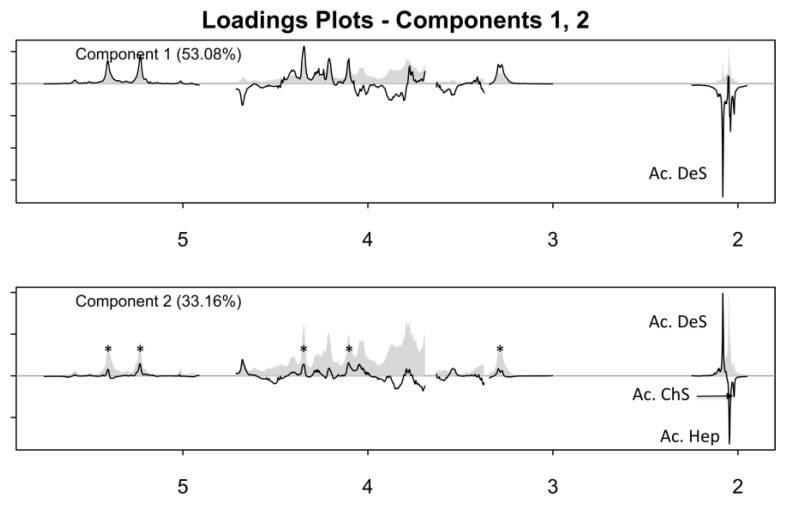
Loading plot of the first two components of the PCA of the GAGs signals region. In the loading plot of component 1, a negative signal corresponding to the *N*-acetyl residue of dermatan is observed. Positive signals corresponding to the trisulfated disaccharide (*) (-I2S-GlcNS,6S-) and negative signals corresponding to the *N*-acetyl residue of glucosamine (Hep) and galatosamine (ChS) are observed in the loading plot of component 2.

In the loading plot of the first principal component, the negative signal of the acetyl protons of dermatan sulfate was observed (Figure 3). By contrast, in the loading of the second principal component, signals belonging to the trisulfated disaccharide (corresponding to the most abundant dissacharide of heparin chains) are positive, while the other heparin and chondroitin acetyl signals are negative. The analysis of the loading plots suggested that samples having a positive score in component 1 (Figure 2, group A) contain a low amount of dermatan sulfate, while those having negative score in component 2 (Figure 2, group C) contain highly acetylated heparin with a larger amount of chondroitin. The samples contained within the group B have instead a higher degree of sulfation, together with a large amount of dermatan. The proton spectra of the samples belonging to these groups confirm what was observed by PCA, showing that the spectra of samples belonging to the group A, in the absence of dermatan and chondroitin acetyl signals, match with those of purified heparin (Figure 1).

**Figure 4:**
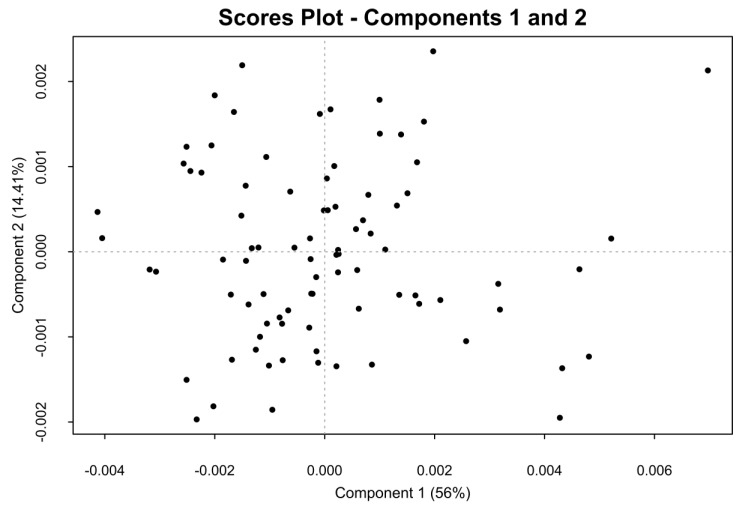
Score plot of the first two components generated by PCA of part of anomeric signals region. No clusters are defined. Only one sample appears isolated in the upper-right of the figure.

Since the major differences among the crude samples defined by PCA are due to the content of galactosaminoglycan and the acetylation degree of the heparin component, the PCA was performed also on part of the anomeric region of the proton spectra (5.75–4.94 ppm). In this region, with the exclusion of the weak H1 of I2S of dermatan sulfate (5.18 ppm), no signals belonging to dermatan and chondroitin are present. Before the PCA analysis, the selected region of the proton spectra was aligned with a custom algorithm (Table S14 and Figure S9), because without alignment the shift effect was dominant in the loading plot of the first principal component. After the alignment, PCA differentiates samples on the basis of the sulfation pattern of the heparin/heparan sulfate components. The score plot generated by the PCA of this spectral region is shown in Figure 4. No evident clusters are visible, and the loading plot of the first principal component (Figure S3) can be interpreted in terms of the degree of sulfation of the samples, where the samples positioned in the negative score of the first principal component are more highly sulfated. Although the loading plot of the second component is difficult to interpret, the negative signals corresponding to the H1 of GlcNS,6OH and I2S linked to GlcNS,6OH indicate that the samples with lower 6-*O*-sulfation are located in the negative score of the second principal component (Figure S3).

### 2.3. SAX-HPLC Analysis

**Figure 5:**
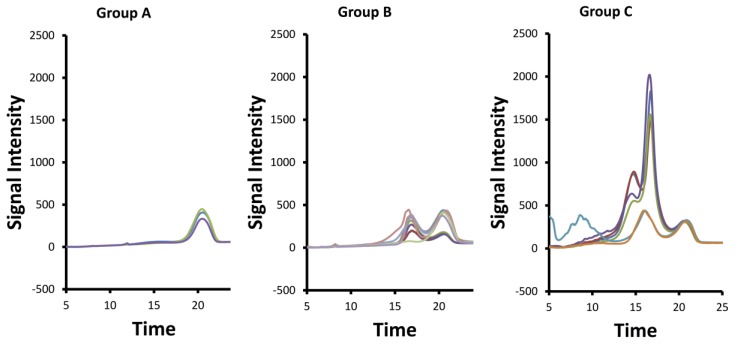
The 5 min to 25 min portion of the strong anion exchange HPLC (SAX-HPLC) chromatograms with UV detection at 215 nm of crudes differentiated into groups A, B, and C by NMR data as shown in Figure 2. Generally, the lower resolution HPLC method shows these groups have increasing levels of different impurities, going from group A to B to C, respectively.

To confirm the results of the NMR chemometric analysis data, the purity of the crude heparin samples was analyzed with a lower resolution method; SAX-HPLC. The SAX-HPLC method applied here separates heparin (~2.5 sulfates per disaccharide) from less sulfated (e.g., ~1 sulfate per disaccaride, DS/CSA/heparan) or more sulfated (e.g., 4 sulfates per disaccharide, over sulfated chondroitin sulfate (OSCS)) GAGs based on negative charge [9,14]. As shown in Figure 5, the crude heparins in group A were the most pure, with the majority of the signal eluting at ~20.5 min consistent with heparin sodium API. For group B, two major peaks (17.0 and 20.4 min) of similar intensity were observed and attributed to primarily DeS and heparin, respectively. For group C, three major peaks were observed (14.5, 16.6, and 20.5 min), with the intensity of the 14.5 and the 16.6 peaks being much higher than the heparin peak at 20.5 min. These group C peaks are attributed to DNA/RNA, heparan/CSA, DeS, and heparin sulfate. Thus, group C samples contain DNA/RNA impurities as well as more less sulfated GAGs (i.e., CSA/heparan/DeS) than the group B or group A samples (Figure S4). Overall, the chemometric analysis of the NMR data and the visual examination of the SAX-HPLC chromatograms of the crude heparin samples can be used to order the crudes from highest (A) to lowest purity (C) as A, B, and C, respectively.

### 2.4. HSQC Analysis

Quantitative HSQC was recently applied to heparin and low molecular weight heparins (LMWHs). The ^1^H,^13^C-HSQC data acquired on heparin APIs were used for calculating the percentage of mono- and disaccharides by normalizing volumes with reference to the sum of volumes of signals corresponding to each monosaccharide type (glucosamines or uronic acids) and the same carbon-proton pair type (e.g., anomeric or C2 position pairs) [10,15,16,17]. In the HSQC spectra collected here, signals belonging to high molecular weight species, mainly DNA and RNA, were not detected, presumably due to the increased linewidths associated with these slower tumbling larger molecules.

**Figure 6:**
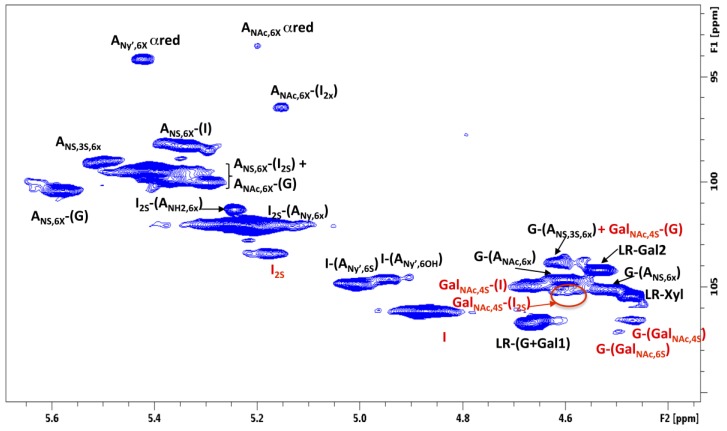
Anomeric region of a crude heparin HSQC spectrum. Signal assignments of heparin and DeS/ChS components are in black and red, respectively.

The HSQC spectrum of crude heparin also contains signals belonging to galactosaminoglycan components (DS and CSA), which partially overlap with those of the heparin/heparan sulfate (HS) components. By contrast, in the H2/C2 region, signals belonging to *N*-acetyl-glucosamine were completely separated from those of the *N*-acetyl-galactosamine residues (3.90 ppm/56.0–56.6 ppm and 4.02 ppm/53.8–54.6 ppm, respectively; Figure S5). Thus, the percentage of glucosamine and galactosamino residues could be calculated by the integration of the corresponding residues (Table 2). In addition, the uronic acid components of the galactosaminoglycans were well separated in the anomeric region of the crude heparins (Figure 6). Beside the major IdoA-GalNAc,4S sequences, DeS contains about 10% of disulfated disaccharide sequences, mostly consisting of IdoA2S-GalNAc,4S [18]. The anomeric signals of IdoA and IdoA2S of dermatan sulfate, at 4.88/106.1 ppm and 5.18/103.4 ppm, respectively, could be integrated to determine the IdoA2S/IdoA ratio. Similarly, the anomeric signal of the glucuronic acid of the chondroitin presents distinct chemical shift values on the basis of the position of the sulfate group of the following *N*-Acetyl-galactosamine residue (Figure 6) (4.47/106.6 ppm and 4.50/107.1 ppm for the ChS4S or ChS6S, respectively) [19], allowing the evaluation of the GalNAc,6S/GalNAc,4S ratio. Unfortunately, the anomeric signals of the DeS and ChS *N*-acetyl-galactosamine residues partially overlap with the anomeric signal of heparin glucuronic acid. In particular, if chondroitin is present, the amount of glucuronic acid linked to trisulfated glucosamine (G-A_NS,3S,6X_) could be overestimated.

Finally, the composition of the heparin fraction was obtained as for the API product [10]. Tables S2 and S3 show the signals assignment and the detailed formulas for the calculations. The precision of the method was evaluated using a crude sample positioned close to the origin (0) of the PCA scores plot of Figure 2, therefore having an average composition in DeS/ChS comparable with the whole library of 88 samples. The intermediate precision was determined combining two operators and two spectrometers operating at 500 and 600 MHz (Tables S7–S9). Using a reasonable experimental time (6 h at 600 MHz and 8 h at 500 MHz) it was possible to quantify with sufficient precision all of the fragments above 3%, compared to 2% for API heparin with 2 h at 600 MHz and 3½ h at 500 MHz [10]. This was considered a good result also in view of the fact that the concentration in the NMR tube was limited to 20 mg/0.6 mL compared to 35 mg/0.6 mL for API heparin. Clearly, the limit of quantification (LOQ) of heparin fragments might change according to the dermatan and chondroitin content. It was verified that a reliable estimation of the LOQ is given by the formula

LOQ = 2000 × VOL/(VOL_SUM × SNR)(1)

where VOL_SUM is the sum of all heparin anomeric signals, while VOL and SNR are the volume and signal to noise ratio of the anomeric signal of I-(A_NY’,6S_) or I-(A_NY’,6OH_) (Figure 6; both residues give comparable values for LOQ). If the LOQ is too high, the spectrum can be acquired again with more scans (NS) for better SNR, as the SNR term is proportional to the square root of NS. The robustness of the method was also evaluated by identifying the most critical parameters of the analytical method: pH of buffer, sample concentration, acquisition temperature, and spectrum phasing. These four parameters where studied at two levels in a Plucket–Burman design (Tables S4–S6). The heparin fragment quantification showed good robustness with respect to all of the parameters. The GAGs content was sensitive to variations of temperature, probably due to the different mobility of the heparin, DeS, and ChS chains.

A summary and the details of the obtained composition of a series of 75 out of 88 crude heparin samples are shown in Table 2 and Tables S10–S13, respectively.

As already observed in the PCA, crude heparin samples are characterized by a large variation in galactosaminoglycan content (0–20%), while the heparin component varies in *N*-acetylation, 6-*O*-sulfation, and nonsulfated uronic acid content (11–21%, 63–79%, and 23–35% respectively). Notably, the serine of the linkage region is almost exclusively present in its intact form (CHα at 4.00/57.4 ppm; Figure S5). However, the oxidized form of serine [20] was detected in the samples of group A by the reduction or absence of the CHα signal and the presence of the anomeric signal of xylose linked to oxidized serine residue (4.48/105.5 ppm; Figure S6). The presence of the oxidized forms in the linkage region suggests that crude heparin has been subject to an oxidizing process; usually, such a step is applied during the purification of crude to API heparin. In fact, the monosaccharide composition of the samples present in group A of the PCA results is compatible with a semi purified product. In addition to showing serine in the oxidized form, their spectra do not contain signals belonging to galactosaminoglycan or DNA/RNA impurities, making the whole spectral profile similar to that of an API product (Figure S6).

### 2.5. Crude of Different Origin

**Figure 7:**
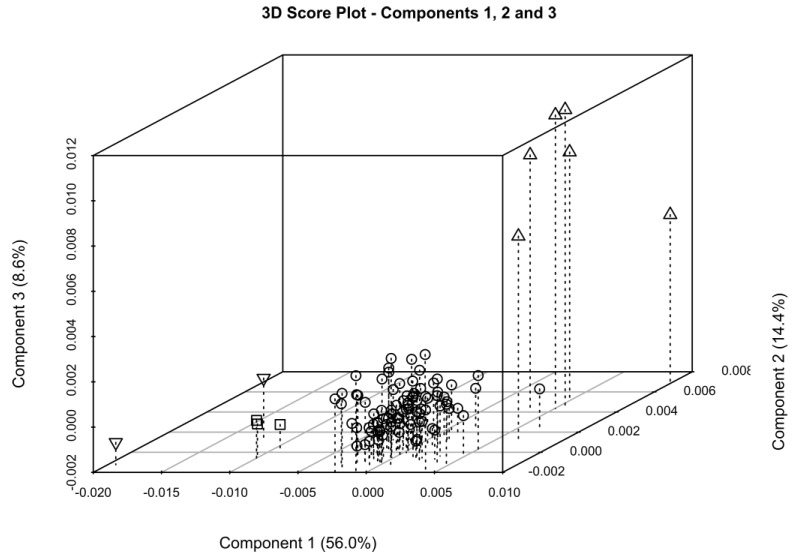
Three-dimensional (3D) Score plot generated by PCA of part of the anomeric signals region. Sample clusters corresponding to the different families of crude heparin are defined. BMHC (above pointing triangles), OMHC (squares) and BLHC (below pointing triangles), against the library of PMHC (circles).

As porcine, bovine, and ovine API heparins differ in many aspects of their structure and activity [21,22,23,24,25], it is important to verify if their structural differences can be observed in the corresponding crude. Six heparin crude samples from bovine intestinal mucosa (BMHC), two from bovine lung (BLHC) and three from ovine intestinal mucosa (OMHC) were characterized by proton and HSQC analysis. The PCA of the GAGs signals region of proton spectra of these samples against the library of porcine crude heparin was not able to differentiate samples according their origin (Figure S7). In spite of the structural differences between the heparins in these crudes, the galactosaminoglycan content mostly contributes to the samples’ separation in component 1 (Figure 3). By contrast, PCA performed exclusively on the anomeric region was able to group samples on the basis of their origin, separating them in function of their sulfation pattern in component 1 and of the 6-*O*-sulfation in component 3 (Figure 7 and Figure S8). Indeed, BMHC and PMHC are not fully separated in components 1 and 2 because of their similar degree of sulfation, while in component 3 BMHC are fully distinguished from other crude heparin (Figure 7).

The monosaccharide composition of each crude family was determined by HSQC analysis (Table 2 and Table 3). The bovine lung crude shows the highest degree of sulfation and lowest content of acetylation and nonsulfated uronic acid. The lower 6-*O*-sulfation of the bovine mucosa crude agree with those observed in the corresponding API [25], while the degree of sulfation and amount of *N*-acetylation measured for the ovine crude samples is intermediate to that of bovine lung and porcine mucosa heparin, as also observed by the integration of C13 spectra of the purified heparin samples [24]. The greater structural detail obtained applying the HSQC method allows the detection of minor residues in crude heparin, such as G2S, that is more abundant in BMHC compared to ovine or porcine heparins [25].

## 3. Discussion

The major goals in the analysis of a large number of porcine crude heparins and a range of crude heparin from different animal or tissue sources were: (1) to establish reliable test methods to differentiate them at the crude stage of manufacturing; and (2) to propose a definition for crude heparins as a key intermediate in drug manufacturing. Similar tests can be used to assure the quality of the porcine heparin or bovine heparin API used clinically. Importantly, by checking heparin quality earlier in the supply chain, contaminated material can be prevented from reaching the active pharmaceutical ingredient purification processes, which are often performed under cGMP in U.S. based plants. Because the supply chain for heparin is complex and a global one, controlling the quality of this widely used drug, its intermediates, and starting materials (at least as early as the crude heparin warehouse) solely through cGMP inspection is insufficient and often impractical, in part due to the limited resources that the regulatory authorities have. Thus, there is a need to develop strong analytics and appropriate specifications for crude heparin to ensure the consistent high quality of the final heparin drug substance. For this reason, the tests and the data obtained by applying them will help assure the quality of heparin sodium and LMWH drug products by improving the quality of the entire global heparin supply chain.

In this work, strong anion exchange HPLC (SAX-HPLC) and NMR spectroscopy coupled with chemometrics was demonstrated to be a feasible strategy to characterize crude heparin samples. The proton NMR and HPLC data clearly show that crude heparins vary in terms of glycosaminoglycan composition, amount of DNA/RNA, and other impurities. Proton spectra, when the samples were properly prepared, could be analyzed by PCA, grouping samples based on their composition (relative amount of dermatan, chondroitin, and heparin) or structural properties (degree of sulfation). The presence in the library of some samples that were almost free from galactosaminoglycans and DNA/RNA components suggests that some batches have undergone different levels of purification treatments consistent with the known differences in the way heparins are manufactured by different companies. Quantitative HSQC analysis, recently applied to determine the mono- and disaccharide composition of heparin and LMWH [10], can be extended to crude heparin, allowing an in-depth study of the structural features of samples. Particularly, the degree of sulfation can vary starting from 2.1 to 2.4, the latter value is typical of the purified porcine heparin. Notably, the group of samples that lacked dermatan and chondroitin also contained oxidized serine, suggesting that the products were treated with oxidizing agents, such as those typically used during the production of API. Overall, PCA and HSQC analyses were able to distinguish between crude heparins of different animal and organ sources and manufactured by different processes.

## 4. Methods

### 4.1. Reagents and Starting Material

Deuterium oxide 99.9%, sodium dihydrogenphosphate hydrate (NaH2PO4 H2O), disodium hydrogen phosphate dihydrate (Na_2_HPO_4_·H_2_O_2_), and 3-(trimethylsilyl)propionic-2,2,3,3-d4 acid sodium salt (TSP) were purchased from Sigma-Aldrich (Milan, Italy). Deuterated EDTA d-16 98% was obtained from Product Cambridge Isotope Laboratories, Inc.

The crude heparin samples were dissolved in phosphate buffer solution, which was prepared as follows: 49.7 mg of sodium dihydrogenphosphate hydrate (0.36 mmol), 202.9 mg of disodium hydrogen phosphate dihydrate (1.14 mmol), and 9.2 mg deuterated EDTA d-16 (0.03 mmol) were dissolved in 10 mL of water. The pH was checked at 7.1. The solution was distributed into 1.3 mL aliquots and then lyophilized. Each aliquot was dissolved in 0.2 mL of D_2_O and lyophilized again. Finally, the buffer was dissolved in 1.3 mL deuterium oxide with 0.002% TSP (12 mM).

### 4.2. Strong Anion Exchange HPLC:

Solutions of crude heparin were prepared as described in Keire et al. [9]. SAX-HPLC separations were performed on a Dionex IonPac^®^ AS11-HC (250 mm × 4 mm) column (Dionex, Sunnyvale, CA, USA). The AS11-HC column’s characteristics are: a bead diameter of 9 m with a 2000 Å pore size, particles made of a divinylbenzene/ethylvinylbenzene polymer cross-linked at 55%, coated with microporous latex (DVB/EVB 6% cross-linked) 70 nm particles with hydroxyalkyl quaternary ammonium functional groups, and capacity of 290 equiv./4 mm × 250 mm column. A column temperature of 35 °C was used. The mobile phase was MilliQ water (buffer A) and 2.5 M NaCl with 20 mM TRIS adjusted to pH 3.0 by addition of phosphoric acid (buffer B). The gradient was 0–2 min at 95% A with 5% B, followed by a linear gradient to 100% B at 26 min, a hold at 100% B until 31 min, a linear gradient to 95% A with 5% B at 32 min, and a hold until end of run at 40 min. The flow rate was constant at 0.8 mL/min. The UV detector was set at 215 nm. A 40 L injection volume was used. The liquid chromatography system consisted of Agilent HPLC with a G1314A variable wavelength detector, G1322A degasser, G1311A quaternary pump, column thermostat, and G1313A autosampler.

### 4.3. Sample Preparation

A 60 mg aliquot of each sample was dissolved in 1.5 mL of D_2_O. After two hours, the samples were centrifuged for 4′ at 12,000 rpm, and the supernatant was separated from the precipitated pellet. The supernatant and precipitated pellet were lyophilized. A 20 mg aliquot of the lyophilized supernatant powder was dissolved in 0.6 mL of phosphate buffer solution and transferred to a 5 mm NMR tube.

### 4.4. ^1^H-NMR

NMR spectra were measured on a Bruker AVANCE III 600 MHz spectrometer (Karlsruhe, Germany) equipped with TCI 5 mm cryogenic probe. The experiments were acquired with a constant presaturation power of 7 Hz at 298 K, and the following acquisition parameters were used: number scan 32, dummy scan 8, relaxation delay 12 s, spectra width 18 ppm, transmitter offset 4.7 ppm. After exponential multiplication (line broadening of 0.3 Hz), the spectra were Fourier transformed, phased, baseline corrected, and calibrated on the TSP signal.

### 4.5. HSQC-NMR

The 2D-^1^H,^13^C-HSQC spectra were measured on a Bruker AVANCE III 600 MHz spectrometer or on a Bruker AVANCE III HD 500 MHz spectrometer (Karlsruhe, Germany), equipped with TCI 5 mm cryogenic probes, using the Bruker library hsqcetgpsisp2.2 pulse sequence.

The experiments were recorded at 298 K using the following acquisition parameters: number of scan 24 (600 MHz) or 40 (500MHz), dummy scan 16, relaxation delay 2.5 s (600 MHz) or 2 s (500 MHz ), spectral width 8 ppm (F2) and 80 ppm (F1), transmitter offset 4.7 ppm (F2) and 80 ppm (F1), 1JC-H = 150 Hz. The chosen processing parameters are: zero filling to 4k in F2; linear prediction to 640 points and zero filling to 1k in F1; and apodization by a 90° shifted squared sine bell function in both dimensions. The spectra were processed and integrated using Topsin software version 3.5 (Bruker BioSpin, Rheinstetten, Germany).

### 4.6. PCA

Proton NMR spectra were imported into R (R Core Team (2016). R: A language and environment for statistical computing. R Foundation for Statistical Computing, Vienna, Austria. URL https://www.R-project.org/) (R version 3.3.1) and cut according to the chosen spectral region (GAGs signals region: 1.95–2.25, 3.0–3.345, 3.37–3.63, 3.69–4.714, and 4.912–5.75 ppm regions, or part of the anomeric signals region: 4.938–5.75 ppm region). Bucketing was then applied (16 points for GAGs region and 8 points for Heparin/HS region). Spectra were normalized for total area, in the Heparin/HS region case aligned (details in Table S14 and Figure S9), and mean centered. Principal Components Analysis (PCA) was performed using the prcomp function from the R stats package.

## Figures and Tables

**Table 1:** Recovering of soluble and insoluble material obtained by two crude heparin samples. Weightings before and after the separation and lyophilization procedure are shown.

Sample	Crude (mg/mL)	Stirring	Supernatant (mg)	Precipitate (mg)	Weight loss % (%w)	Precipitate (%w)
**G9709**	40.4	No	35.3	1.2	9.7	3.3
**G9709**	40.8	No	35.4	1.0	10.8	2.7
**G9709**	38.9	No	33.7	1.2	10.3	3.4
**G9709**	39.0	Yes	34.0	1.1	10.0	3.1
**G9709**	79.7	No	60.4	10.0	11.7	14.2
**G9709**	81.4	Yes	62.4	9.4	11.8	13.1
**G9710**	39.0	No	33.8	2.7	6.4	7.4
**G9710**	39.3	Yes	33.2	2.1	10.2	5.9
**G9710**	79.5	No	56.8	13.8	11.2	19.5
**G9710**	80.5	Yes	59.3	13.0	10.2	18.0

**Table 2:** Molar average composition of 88 crude heparin samples. PMHC = pig mucosa heparin crude; Hep = Heparin; DeS = Dermatan sulfate; ChS = Chondroitin sulfate; A = Glucosamine; I = Iduronic acid; G = Glucuronic acid; X = SO_3_*^−^* or H; SDEG = Heparin disaccharide degree of sulfation.

PMHC	Hep	DeS	ChS	ANH26X	ANAc6X	ANS3S6X	A6S	G2OH	G2S	I2OH	I2S	SDEG
**average**	89.3	8.6	2.4	1.8	16.3	4.5	73.3	18.0	0.01	10.5	71.5	2.31
**median**	89.2	8.7	2.1	1.9	16.3	4.4	73.7	17.9	0.00	10.6	71.4	2.32
**st.dev.**	4.1	3.1	1.9	0.7	2.0	0.7	3.4	1.9	0.09	0.9	2.5	0.08
**min**	79.9	0.0	0.0	0.0	11.6	3.1	63.3	14.0	0.00	8.2	65.4	2.13
**max**	100.0	16.6	7.7	3.0	21.1	5.9	79.8	22.4	0.75	12.4	76.8	2.46

**Table 3:** Molar average composition of crude heparin of different species/organ.

BMHC	Hep	DeS	ChS	ANH26X	ANAc6X	ANS3S6X	A6S	G2OH	G2S	I2OH	I2S	SDEG
**average**	94.7	4.2	0.4	1.5	10.6	2.0	51.7	14.1	1.5	6.8	77.1	2.20
**median**	98.1	1.1	0.0	1.9	9.6	1.8	52.4	13.8	2.0	6.3	76.9	2.19
**st.dev.**	5.9	5.8	0.9	1.2	3.3	0.7	4.0	2.0	1.2	1.2	2.8	0.07
**min**	85.3	0.0	0.0	0.0	8.2	1.3	47.1	11.9	0.0	6.0	73.4	2.12
**max**	98.8	12.5	2.1	2.6	17.2	3.5	57.2	17.4	2.5	9.2	81.0	2.31
**BLHC**	**Hep**	**DeS**	**ChS**	**ANH26X**	**ANAc6X**	**ANS3S6X**	**A6S**	**G2OH**	**G2S**	**I2OH**	**I2S**	**SDEG**
**Sample 1**	94.7	4.2	0.4	1.9	6.6	2.8	85.8	7.8	0.0	4.6	87.6	2.68
**Sample 2**	98.1	1.1	0.0	0.0	1.6	2.5	90.2	2.3	0.5	2.8	93.4	2.85
**OMHC**	**Hep**	**DeS**	**ChS**	**ANH26X**	**ANAc6X**	**ANS3S6X**	**A6S**	**G2OH**	**G2S**	**I2OH**	**I2S**	**SDEG**
**Sample 1**	98.9	1.1	0.0	1.5	8.8	5.0	80.5	10.2	0.0	7.0	82.8	2.58
**Sample 2**	97.7	2.3	0.0	1.8	8.8	4.7	82.2	10.5	0.0	7.2	82.3	2.59
**Sample 3**	98.7	1.3	0.0	1.6	7.8	5.3	82.2	9.7	0.0	7.0	83.4	2.61

## Supplementary Materials

Figures S1–S9 and Tables S1–S14 are available online
