# Supplementary material for: Combining NMR Spectroscopy and Chemometrics to Monitor Structural Features of Crude Hep-arin
Source: Molecules. 2017 Jul 8;22(7):1146. doi: 10.3390/molecules22071146 (PMC6152302; doi:10.3390/molecules22071146)
Supplement: Supplementary file 1 [file molecules-22-01146-s001.pdf]

Supporting Information

Article

Combining NMR spectroscopy and chemometrics to monitor structural features of crude heparin.

Lucio Mauri[1], Maria Marinozzi[1], Giulia Mazzini[1], Richard E. Kolinski[2], Michael Karfunkle[2], David A. Keire[2], Marco Guerrini[1]*

[1*]Institute for Chemical and Biochemical Research G. Ronzoni, via G. Colombo 81, 20133 Milan, Italy

[2] Division of Pharmaceutical Analysis, Office of Testing and Research, Center for Drug Evaluation and Research, U.S. Food and Drug Administration, 645 S. Newstead Ave., St. Louis, MO 63110, USA

*****Correspondence: guerrini@ronzoni.it; Tel.: +39-02-70641621

List of Figures

- **Fig. S1.** ^{1}H spectra of the supernatant and re-suspended precipitate.
- **Fig. S2.** ^{1}H spectra of crude heparin samples in D_2O and phosphate buffer.
- **Fig. S3.** Loading plot of the first two principal components of PCA of the anomeric region of PMHC spectra.
- **Fig. S4.** ^{1}H spectra of crude heparin samples belonging to group A, B and C.
- **Fig. S5.** HSQC spectrum of crude heparin – ring signals region.
- **Fig. S6.** HSQC spectrum of crude heparin – anomeric signals region.
- **Fig. S7.** Score plot of the first two components of the PCA of the glycosaminoglycan regions, showing samples of crude heparin from different origins.
- **Fig. S8.** Loading plots of the first and third component of the PCA of the anomeric region of PMHC spectra.
- **Fig. S9.** Results of the alignment algorithm.

List of Tables

- **Table S1.** Summary results of preparation procedure.
- **Table S2.** HSQC Integration regions.
- **Table S3.** Formulas for the HSQC quantification.
- **Table S4.** Plackett-Burman design for the HSQC robustness study.
- **Table S5.** Results of the HSQC quantification for the Plackett-Burman design.
- **Table S6.** Effects of factors of the Plackett-Burman design.
- **Table S7.** Experimental design for the HSQC intermediate precision study.
- **Table S8.** Results of the HSQC quantification for the intermediate precision experiments.
- **Table S9.** Results of the HSQC intermediate precision study.
- **Table S10.** HSQC molar percentages of glucosamine residues of 75 porcine crude heparin samples.
- **Table S11.** HSQC molar percentages of uronic acids residues of 75 porcine crude heparin samples.
- **Table S12.** HSQC percentages of GAGs of 75 porcine crude heparin samples.
- **Table S13.** Linkage region and sulfation degree of 75 porcine crude heparin samples.
- **Table S14.** Alignment algorithm.

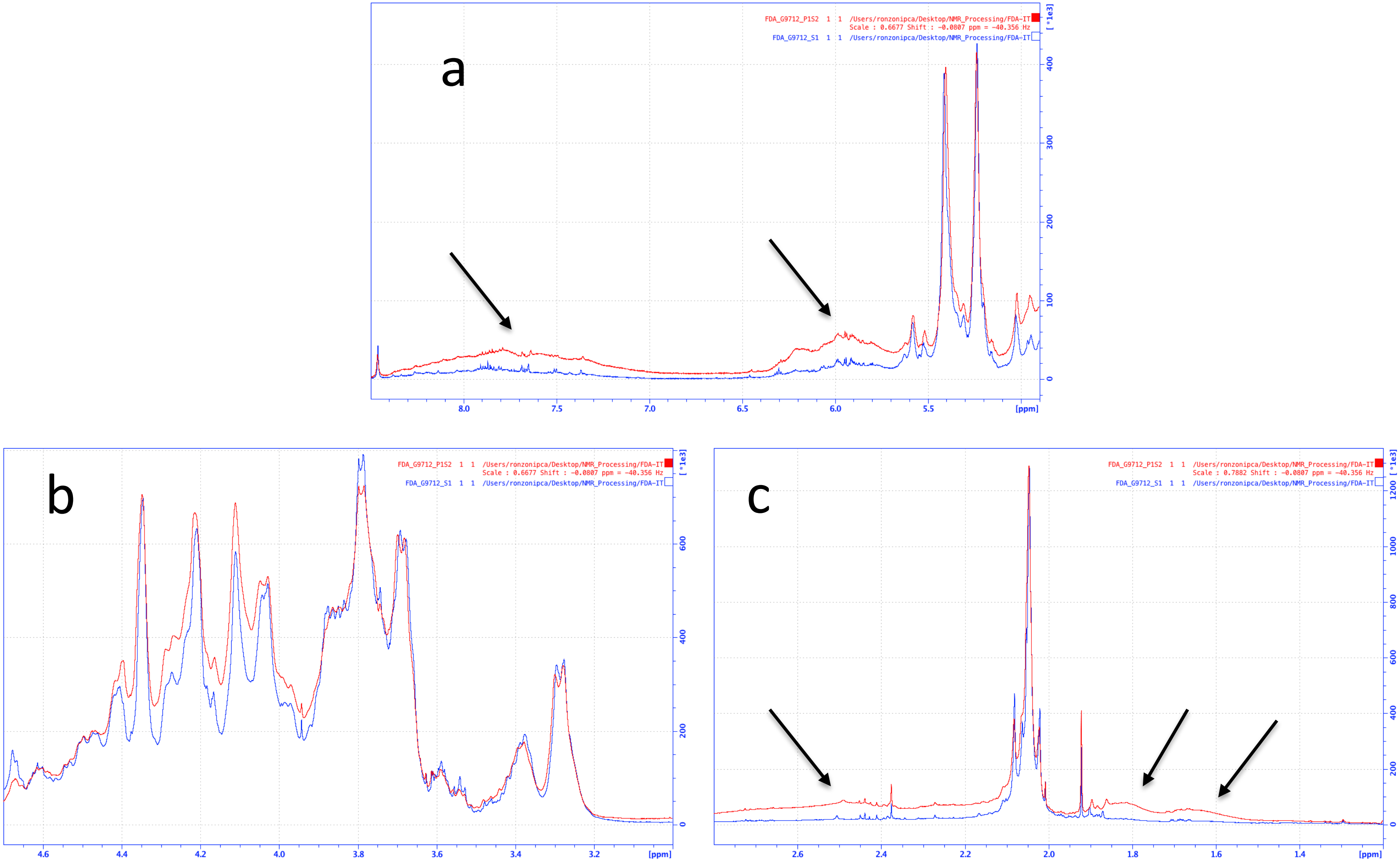

Fig. S1. Low field (a) middle field (b) and high field (c) ^{1}H spectra of crude heparin samples. The spectrum of the supernatant and re-suspended precipitate are shown in blue and red, respectively. Arrows indicate broad signal due to nucleic acid material.

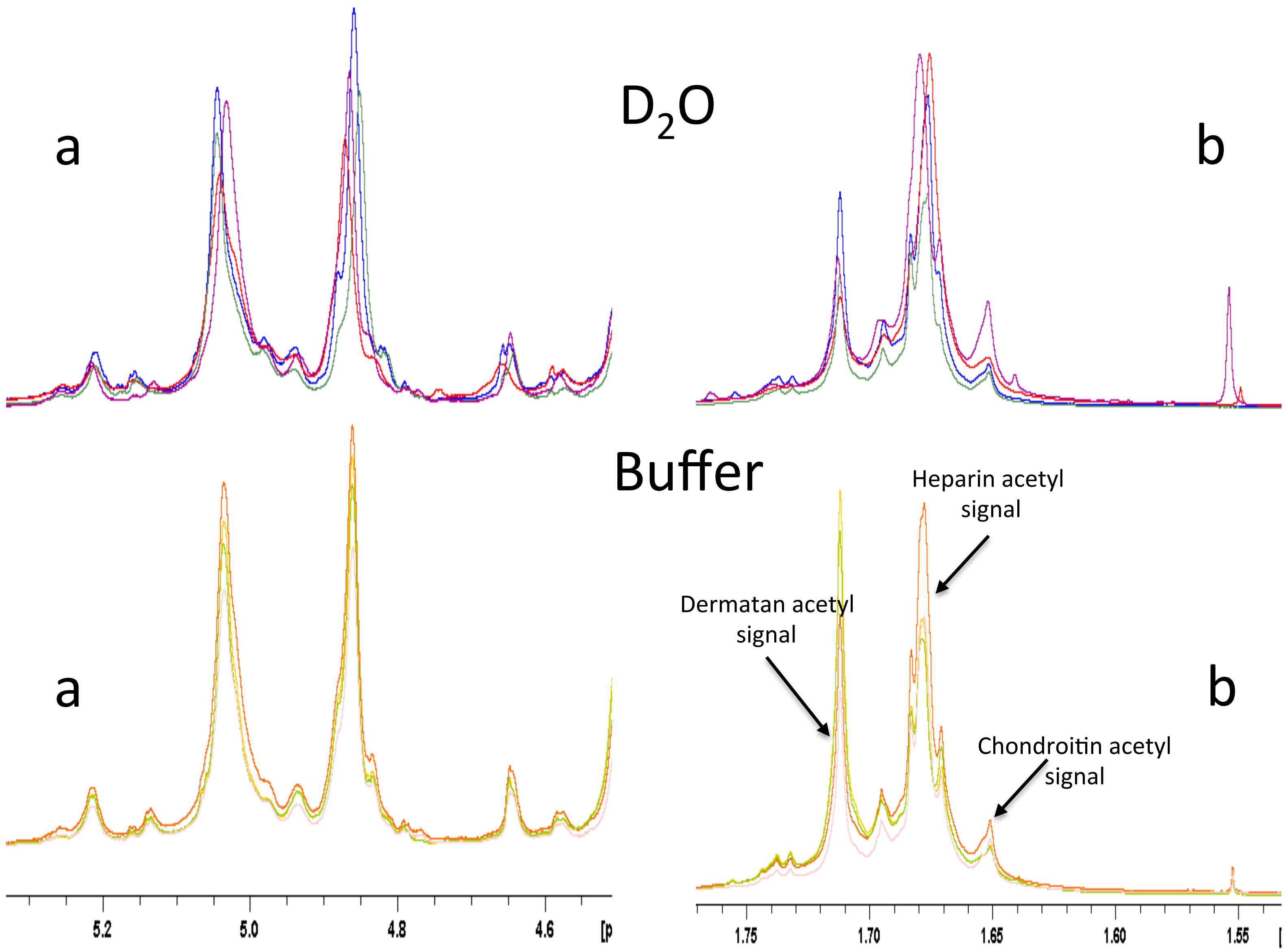

Fig. S2. Anomeric region (a) and acetyl region (b) of four ^{1}H spectra of crude heparin samples in D_2O and phosphate buffer, respectively. Arrows indicate the acetyl signals of heparin, dermatan and chondroitin components.

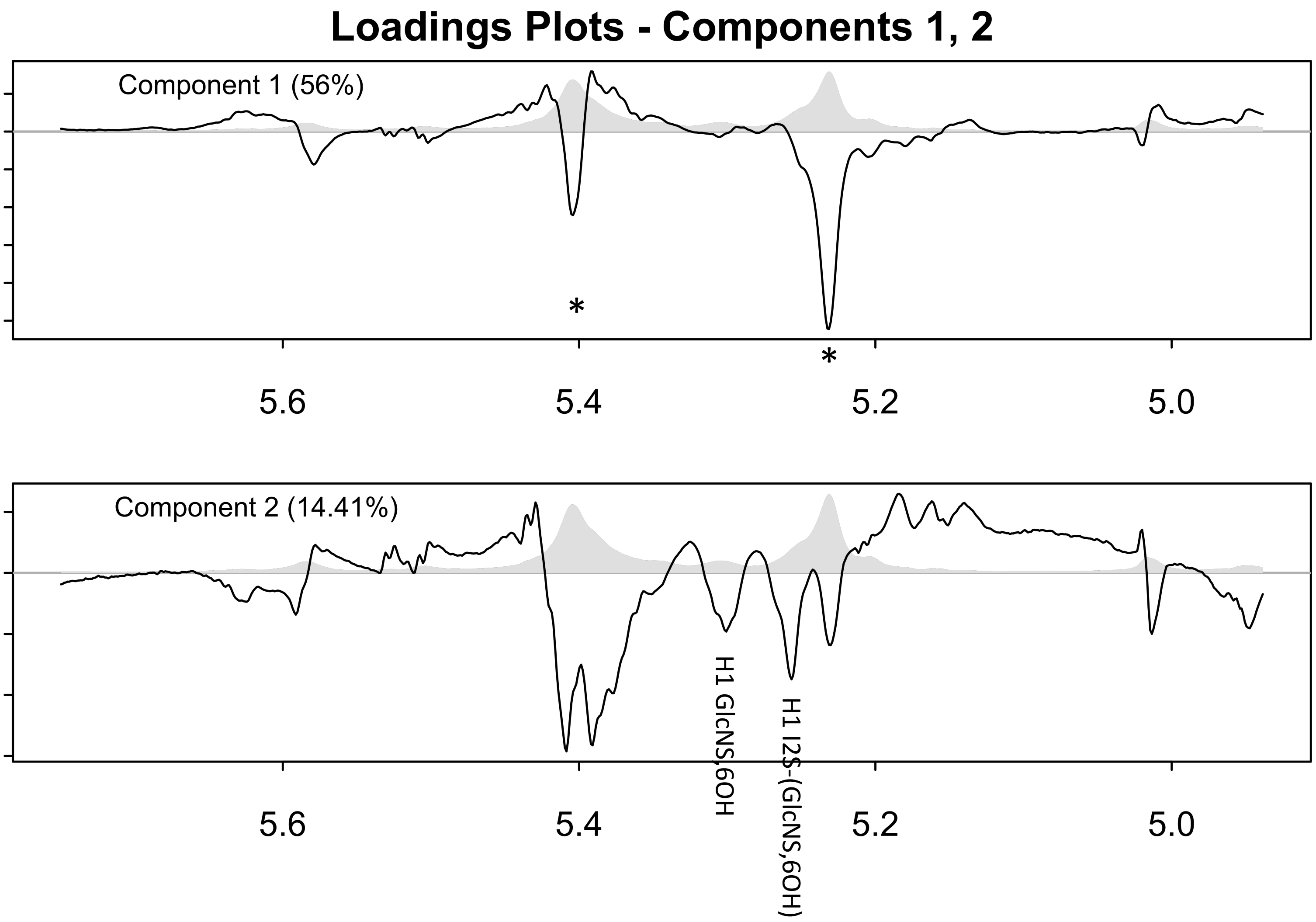

Fig. S3. Loading plot of the first two components derived from the PCA of part of the anomeric region. In the loading plot of component 1, negative signals corresponding to the trisulfated disaccharide (*) (-I2S-GlcNS,6S-) are observed. Negative signals corresponding to the H1 of GlcNS,6OH and I2S linked to GlcNS,6OH are observed in the loading plot of the component 2.

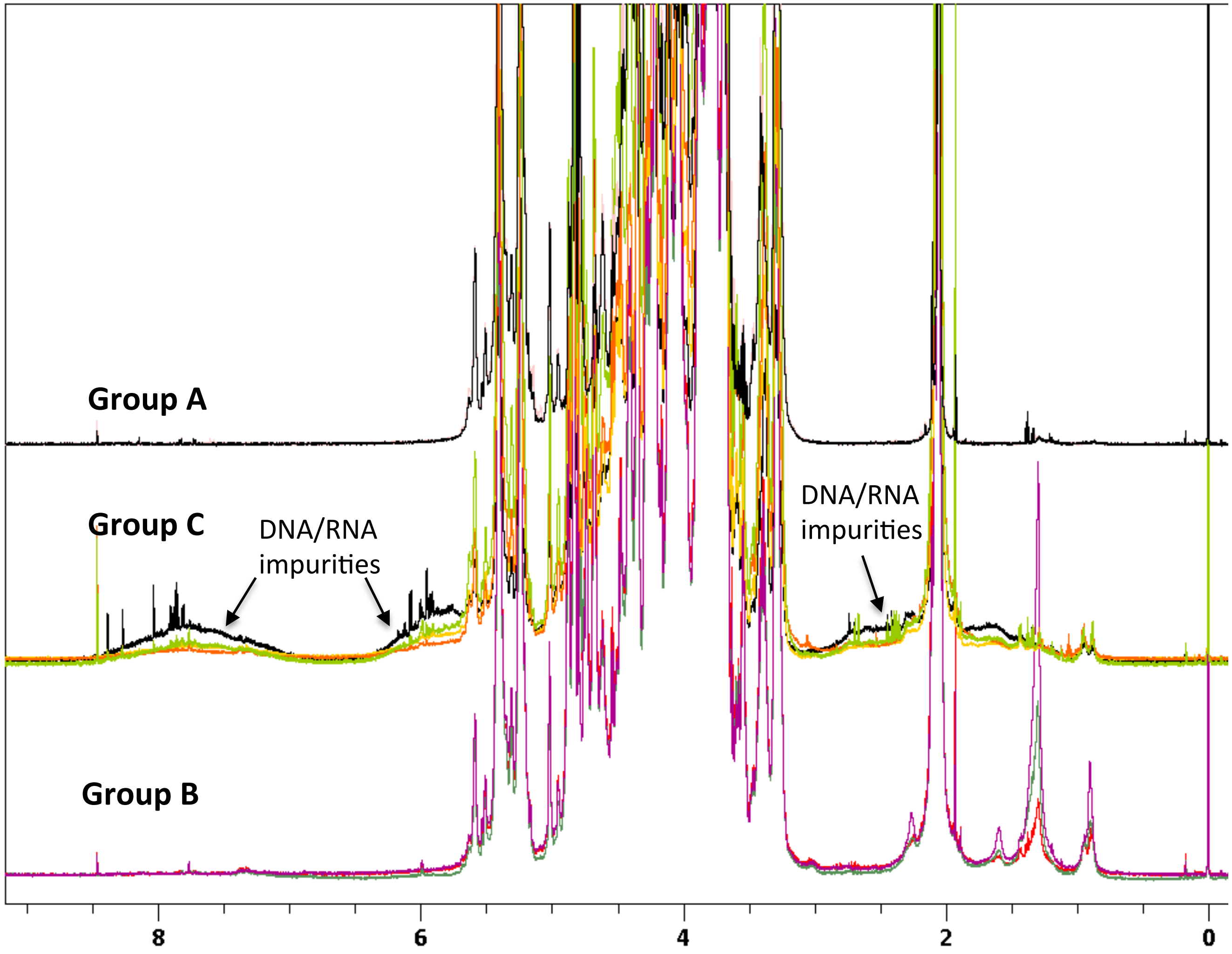

Fig. S4. ^{1}H spectra of crude heparin samples belonging to group A, B and C.

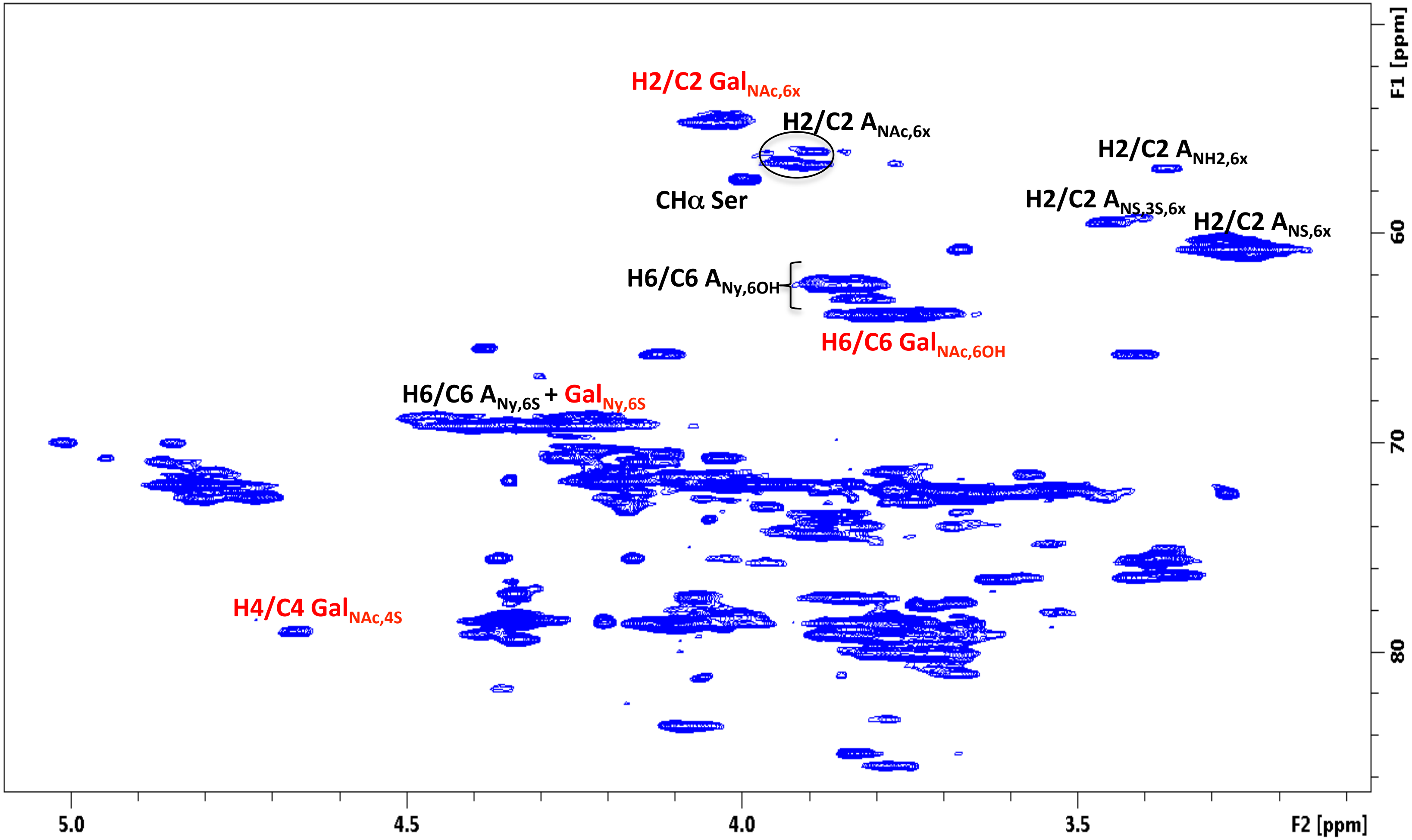

Fig. S5. Ring region of the HSQC spectrum of crude heparin. Signal assignments of heparin and DeS/ChS components are in black and red, respectively.

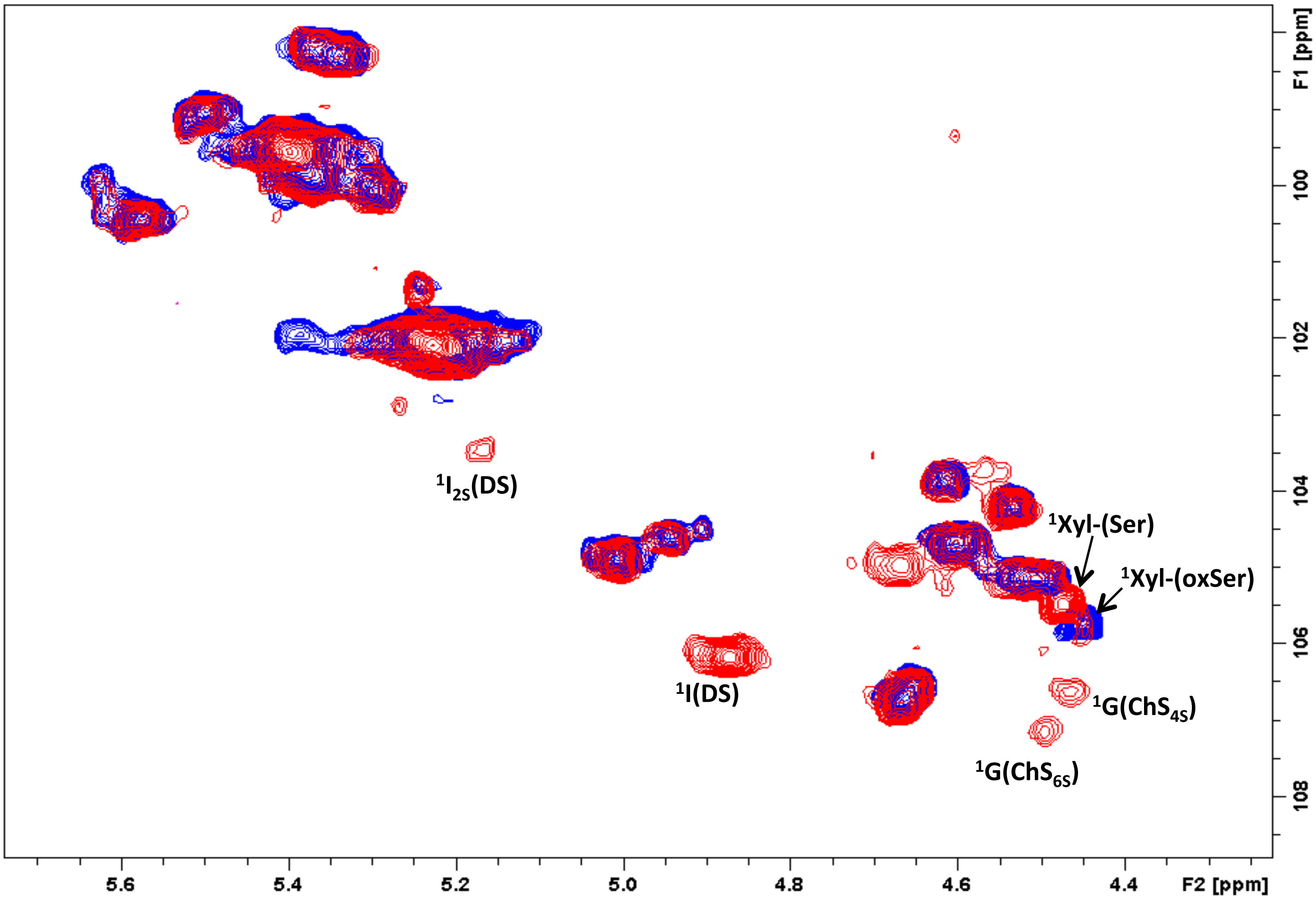

Fig. S6. Anomeric region of the HSQC spectra of crude heparin samples. Overlay of an HSQC spectrum of a crude heparin sample with 100% oxidized serine (blue) and one without oxidation (red).

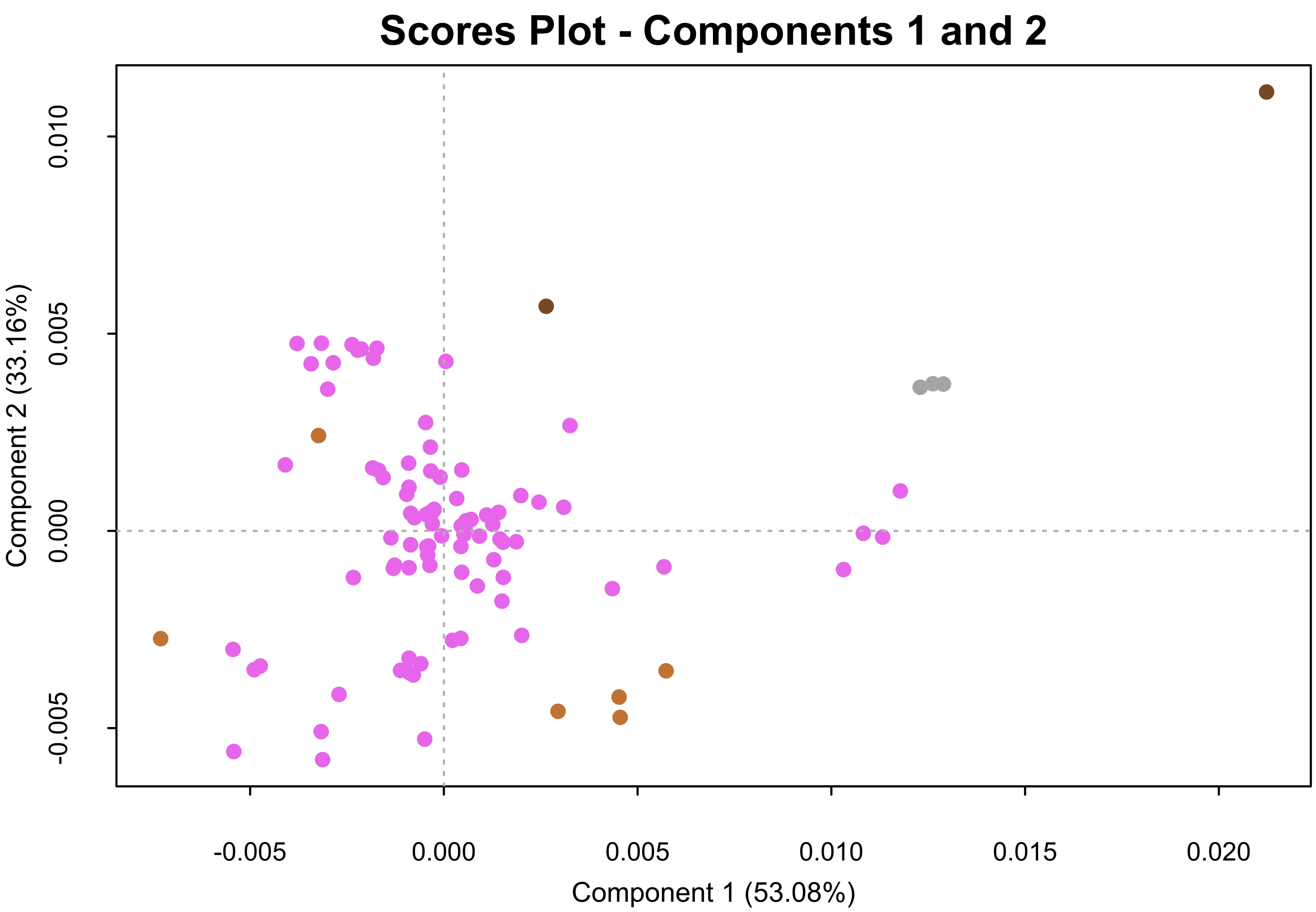

Fig. S7. Score plot of the first two components generated by PCA of BMHC (brown), OMHC (gray) and BLHC (dark brown), against the library of PMHC (violet). Glycosaminoglycan regions of proton spectra were used.

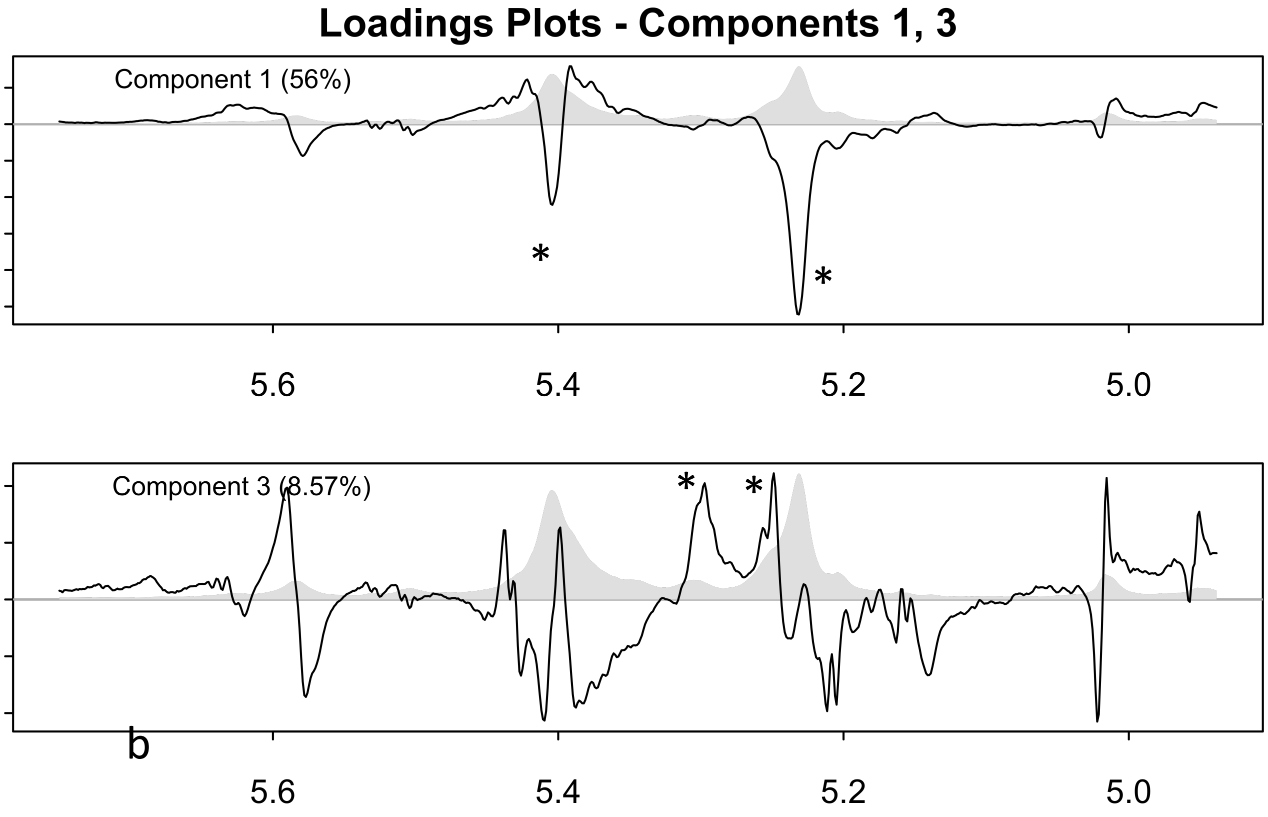

Fig. S8. Loading plots of the first and third component of the PCA of the anomeric region of PMHC spectra. Asterisks indicate the signals corresponding to the H1 of GlcNS,6S-IdoA2S disaccharide in the loading of the component 1 (negative) and H1 GlcNS,OH-IdoA2S disaccharide in the loading of the component 3 (positive).

	Weight loss %	Precipitate %	Solubilized %
**mean**	8.8	5.6	85.6
**median**	9.7	4.2	86.4
**stdev**	4.4	4.7	5.8
**CV%**	49.7	84.1	6.8
**MIN**	0.0	0.0	64.6
**MAX**	20.5	27.6	96.1

Table S1. Summary results of preparation procedure on 88 samples (weight loss after lyophilization, soluble and insoluble components).

N	Comp	C#	Name in text	Name in formulas	F1		F2	
1	HEP	2	$^{2}A_{NS,6X}$	2ANS6X	62.28	59.79	3.40	3.14
2	HEP	2	$^{2}A_{NS,3S,6X}$	2ANS3S6X	59.95	58.84	3.51	3.36
3	HEP	2	$^{2}A_{NAc,6X}$	2ANAc6X	57.14	55.76	4.02	3.83
4	HEP	2	$^{2}A_{NH2,6X}$	2ANH26X	57.26	56.59	3.42	3.35
5	HEP	6	$^{6}A_{NY,3X}$	6ANY3X	63.46	61.80	3.99	3.74
6	HEP	6	$^{6}A_{NY,3X,6S}$	6ANY3X6S	70.06	68.29	4.59	4.11
7	HEP	1	$^{1}A_{NS,6X}$-(G)	1ANS6X_G	100.99	99.74	5.66	5.52
8	HEP	1	$^{1}A_{NS,3S,6X}$	1ANS3S6X	99.32	98.69	5.56	5.49
9	HEP	1	$^{1}A_{NS,6X}$-(I_{2S}) + $^{1}A_{NAc}$-(G)	Atot	100.81	99.01	5.48	5.26
10	HEP	1	$^{1}A_{NS,6X}$-(I)	1ANS6X_I	98.75	97.88	5.43	5.30
11	HEP	1	$^{1}A_{NAc,6X}$-(I)	1ANAc6X_I	96.85	96.22	5.17	5.13
12	HEP	1	$^{1}A_{NS,6X}$αRed	1ANS6X_aRed_X	94.37	93.56	5.48	5.42
13	HEP	1	$^{1}A_{NAc,6X}$αRed	1ANAc6X_aRed	93.72	93.14	5.23	5.20
14	HEP	1	$^{1}A_{NS,6X}$-(GalA) + Unk	1ANS6X_GalA_Unk	102.41	101.54	5.43	5.38
15	HEP	1	$^{1}I_{2S}$-($A_{NY',3X,6X}$) + ^{1}GalA	I2Stot	102.73	101.64	5.34	5.05
16	HEP	1	^{1}I-($A_{NY',6S}$)	1I_ANY6S	105.34	104.51	5.06	4.97
17	HEP	1	^{1}I-($A_{NY'}$)	1I_ANY	104.99	104.33	4.97	4.92
18	HEP	1	$^{1}G_{X}$	1GX	103.95	103.37	4.72	4.68
19	HEP	1	^{1}G-($A_{NS,3S,6X}$)	1G_ANS3S6X	104.19	103.56	4.66	4.58
20	HEP	1	^{1}G-($A_{NS,6X}$)	1G_ANS6X	105.32	104.50	4.68	4.56
21	HEP	1	^{1}G-($A_{NAc,6X}$) + ^{1}Xyl-(Ser)	1G_ANAc6X	105.73	104.84	4.56	4.45
22	HEP	1	^{1}Gal-(Xyl)	1Gal_Xyl	104.46	104.00	4.57	4.50
23	HEP		α Serine	1Xyl_Ser	57.69	57.04	4.03	3.98
24	HEP	1	^{1}Xyl-(oxSer)	1Xyl_oxSer	106.11	105.58	4.48	4.43
25	HEP	1	^{1}G-(Gal) + ^{1}Gal-(Gal)	2LR	107.14	106.22	4.71	4.61
26	HEP	5	^{5}GalA	5GalA	74.85	74.25	4.73	4.67
27	HEP	1	^{1}2,3-epoxide	1epoxide	97.78	97.24	5.45	5.41
28	HEP	1	$^{1}I_{2S}$-($A_{NH2,6X}$)	1I2S_ANH26X	101.54	100.97	5.28	5.22
29	HEP	4	$^{4}G_{NR}$	4GNR	75.24	74.53	3.59	3.51
30	HEP	3	$^{3}G_{NR}$	3GNR	78.60	77.69	3.58	3.51
31	HEP	2	$^{2}A_{NAc}$ox	2ANAc_ox	59.56	58.41	4.42	4.33
32	ChS	1	^{1}G-($GalN_{4S}$)	1G_ChS4S	106.86	106.27	4.50	4.44
33	ChS	1	^{1}G-($GalN_{6S}$)	1G_ChS6S	107.26	106.88	4.52	4.48
34	DS	1	^{1}I-(GalN)	1I_DS	106.54	105.60	4.96	4.82
35	DS	1	$^{1}I_{2S}$-(GalN)	1I2S_DS	103.67	103.04	5.21	5.14
36	DS+ChS	2	^{2}GalN	2GalN_DSChS	55.21	53.07	4.12	3.98
37			noise	noise	59	53	2.0	1.5

(1) Overlapped with GalNAc6S ChS

Table S2. HSQC Integration regions

**1 - *Preliminary calculations:***	
A1SUM	v_1ANS6X_G + v_1ANS3S6X + v_Atot + v_1ANS6X_I + v_1ANAc6X_I2X + v_1ANS6X_aRed_X + v_1ANAc6X_aRed + v_1ANS6X_GalA_Unk + v_1epoxide
A2SUM	v_2ANS6X + v_2ANS3S6X + v_2ANAc6X + v_2ANH26X
A6SUM	v_6ANY3X + v_6ANY3X6S
USUM	v_I2Stot + v_1I2S_ANH26X + v_1I_ANY6S + v_1I_ANY + v_1GX + v_1G_ANS3S6X + v_1G_ANS6X + v_1G_ANAc6X + v_1epoxide
GAGSUM	A2SUM+v_2GalN_DSChS
DCSUM	v_1G_ChS4S + v_1G_ChS6S + v_1I_DS + v_1I2S_DS
**2 - *Percent composition expressed in glycosaminoglycans components:***	
Hep	100 * A2SUM / GAGSUM
DS	IF [DCSUM > 0] 100 * (v_2GalN_DSChS / GAGSUM) * (v_1I_DS+v_1I2S_DS) / DCSUM<br>ELSE 0
ChS	IF [DCSUM > 0] 100 * (v_2GalN_DSChS / GAGSUM) * (v_1G_ChS4S+v_1G_ChS6S) / DCSUM<br>ELSE 0
**3 - *Internal composition of glucosamines in heparin and heparan sulfate, expressed in percent:***	
$A_{NAc,6x}$-(G)	v_2ANAc6X*100 / A2SUM - (v_1ANAc6X_I2X + v_1ANAc6X_aRed)*100 / A1SUM
$A_{NAc,6x}$-(I)	v_1ANAc6X_I2X*100 / A1SUM
$A_{NS,6x}$-(G)	v_1ANS6X_G*100 / A1SUM
$A_{NS,6x}$-(I)	v_1ANS6X_I*100 / A1SUM
$A_{NS,6x}$-(I_{2S})	v_Atot*100 / A1SUM - v_2ANAc6X*100 / A2SUM + (v_1ANAc6X_I2X + v_1ANAc6X_aRed)*100 / A1SUM
$A_{NS,6x}$-(GalA)+ Unk	v_1ANS6X_GalA_Unk*100 / A1SUM
$A_{NS,6x}$-(Epox)	v_1epoxide*100 / A1SUM
$A_{NH2,6x}$	v_2ANH26X*100 / A2SUM
$A_{NAc,6x}$	v_2ANAc6X*100/ A2SUM
$A_{NS,3S,6x}$	v_1ANS3S6X*100 / A1SUM
$A_{NAc,6x}$αRed	v_1ANAc6X_aRed*100 / A1SUM
$A_{NS,6x}$αRed	v_1ANS6X_aRed_X*100/A1SUM - v_2ANH26X*100 / A2SUM
$A_{NS,6X}$	100 - A_NH26X - A_NAc6X - A_NS3S6X
**4 - *Internal composition of uronic acids in heparin and heparan sulfate, expressed in percent:***	
G-($A_{NAc,6x}$)	v_1G_ANAc6X*100 / USUM
G-($A_{NS,6x}$)	v_1G_ANS6X*100 / USUM
G-($A_{NS,3S,6x}$)	v_1G_ANS3S6X*100 / USUM
I-($A_{Ny'}$)	v_1I_ANY*100 / USUM
I-($A_{Ny',6S}$)	v_1I_ANY6S*100 / USUM
I_{2S}-($A_{NH2,6x}$)	v_1I2S_ANH26X*100 / USUM
I_{2S}-($A_{Ny',3x,6x}$)	(v_I2Stot - v_5GalA)*100 / USUM
G_{2OH}	(v_1G_ANS6X + v_1G_ANAc6X + v_1G_ANS3S6X)*100 / USUM
G_{2S}	v_1GX*100 / USUM
I_{2OH}	(v_1I_ANY6S+ v_1I_ANY)*100 / USUM
I_{2S}	(v_1I2S_ANH26X + v_I2Stot - v_5GalA)*100 / USUM
GalA	v_5GalA*100 / USUM
2,3-epoxide	v_1epoxide*100 / USUM
**5 - *% 6S sulfation:***	
% 6S	v_6ANY3X6S*100 / A6SUM

Table S3.1 - Formulas for the HSQC quantification

**6 - *Evaluation of the ligand region and the percentage of oxidized serine:***	
LR	v_2LR*100 / (A1SUM + USUM)
oxSer	IF [v_1Xyl_Ser+v_1Xyl_oxSer > 0] v_1Xyl_oxSer*100 / (v_1Xyl_Ser+v_1Xyl_oxSer)<br>ELSE 'Not Available: Serine not Detected!'
**7 - *Degree of sulfation:***	
DSulf	(A_6S + A_NS6X + 2*A_NS3S6X + G_2S + I_2S) / 100
**8 - *Additional Results:***	
GNR	(v_3GNR + v_4GNR)*100 / (2*USUM)
A_{NAc}-ox	v_2ANAc_ox*100 / A2SUM
$A_{NS,6X}$ from ^{2}A	v_2ANS6X / A2SUM*100
$A_{NS,3S,6X}$ from ^{2}A	v_2ANS3S6X / A2SUM*100
$^{2}A_{SUM}/^{1}A_{SUM}$	A2SUM / A1SUM
$^{1}U_{SUM}/^{1}A_{SUM}$	USUM / A1SUM
Disacch Mass	336 + 23 + SDEG * 102 + A_NAc6X * 42 / 100
**9 - *Sulfation distribution dermatan sulfate and of chondroitin:***	
% 2-sulfated DS<br>(short. DS2S)	IF [v_1I_DS+v_1I2S_DS > 0] 100 * v_1I2S_DS / (v_1I_DS+v_1I2S_DS)<br>ELSE 'Not Available: Dermatan Sulfate not Detected'
% 6-sulfated ChS<br>(short. ChS6S)	IF [v_1G_ChS4S+v_1G_ChS6S > 0] 100 * v_1G_ChS6S / (v_1G_ChS4S+v_1G_ChS6S)<br>ELSE 'Not Available: Chondroitin Sulfate not Detected'

Table S3.2 - Formulas for the HSQC quantification

Design scheme

Factor	Weight	Buffer pH	Temp.	Phase[1]	DM1	DM2	DM3
Low level (-1)	18 mg	7.0	296 K	PCH0-5°	NA	NA	NA
High level (1)	22 mg	7.2	300 K	PCH0+5°	NA	NA	NA
EXP 1	1	1	1	1	1	1	1
EXP 2	1	1	-1	1	-1	-1	-1
EXP 3	1	-1	1	-1	1	-1	-1
EXP 4	1	-1	-1	-1	-1	1	1
EXP 5	-1	1	1	-1	-1	1	-1
EXP 6	-1	1	-1	-1	1	-1	1
EXP 7	-1	-1	1	1	-1	-1	1
EXP 8	-1	-1	-1	1	1	1	-1

Effects formulas

$$E_{ij} = \frac{1}{4} \cdot \sum_{k=1}^{8} lv_{ki} \cdot r_{kj} \qquad E_{lim,j} = \pm \sqrt{\frac{\sum_{i=5}^{7} E_{ij}^2}{3}}.$$

E_{ij} is the effect of factor i on fragment j
lv_{kj} is the value (1 or -1) of the level for the factor j in the experiment k
r_{kj} is the value of the fragment j in the experiment k
$E_{lim,j}$ are the limits outside which an effect on fragment j is considered significant

Table S4 - Plackett-Burman design for the HSQC robustness study and formulas for the computation of the effects of factors.
[1] ±5° in PCH0 is referred to the optimal value found by the operator.

	A_NAc6X_G	A_NAc6X_I	A_NS6X_G	A_NS6X_I	A_NS6X_I_2S	A_NS6X_GalA	A_NS6X_Epox	A_NH26X	A_NAc6X	A_NS3S6X	A_NAc6XaRed	A_NS6XaRed	A_NS6X	A_6S
PB1	14.2	0.8	9.2	9.8	58.2	0.0	0.0	1.8	16.1	4.6	1.1	0.2	77.4	75.2
PB2	14.4	0.6	9.7	8.8	59.0	0.0	0.0	2.0	16.1	4.6	1.1	-0.1	77.3	74.7
PB3	13.1	1.0	10.5	9.4	57.2	1.1	0.0	1.9	15.3	4.8	1.2	-0.1	78.1	73.4
PB4	13.7	0.9	10.4	8.4	57.0	2.1	0.0	2.0	15.8	4.1	1.1	0.3	78.2	74.2
PB5	13.1	0.8	10.4	8.6	58.2	1.7	0.0	2.0	14.8	4.1	1.0	0.2	79.1	74.6
PB6	14.1	0.6	8.9	9.0	60.0	0.0	0.0	2.0	15.4	4.4	0.8	0.2	78.1	74.7
PB7	15.1	0.4	9.2	8.7	58.9	0.0	0.7	2.0	16.2	4.4	0.7	-0.1	77.4	75.8
PB8	13.7	0.7	9.8	8.8	59.3	0.0	0.0	2.0	15.4	4.9	1.0	-0.2	77.7	74.7

	G_A_NAc6X	G_A_NS6X	G_A_NS3S6X	I_A_NY	I_A_NY6S	I_2S_A_NH26X	I_2S_A_NY3X6X	G_2OH	G_2S	I_2OH	I_2S	GalA	Epox
PB1	6.9	6.8	3.6	3.7	7.4	2.2	69.4	17.3	0.0	11.0	71.6	0.0	0.0
PB2	7.2	7.4	3.4	4.0	7.7	2.7	67.7	18.0	0.0	11.7	70.3	0.0	0.0
PB3	6.9	7.2	3.9	3.3	7.8	2.4	68.6	18.0	0.0	11.0	71.0	0.0	0.0
PB4	6.8	7.9	3.6	3.6	7.5	2.5	68.0	18.3	0.0	11.1	70.6	0.0	0.0
PB5	7.0	6.8	3.1	3.1	7.8	1.9	70.4	16.8	0.0	10.9	72.2	0.0	0.0
PB6	6.8	7.2	3.8	3.2	7.1	2.2	69.7	17.8	0.0	10.2	71.9	0.0	0.0
PB7	6.2	8.3	3.1	2.8	7.2	2.4	69.3	17.6	0.0	10.0	71.7	0.0	0.7
PB8	6.9	6.9	3.2	3.1	7.0	2.4	70.5	17.0	0.0	10.1	72.8	0.0	0.0

	Hep	DS	ChS	DS2S	ChS6S
PB1	89.5	8.4	2.0	11.2	9.7
PB2	89.9	8.0	2.1	13.7	17.8
PB3	90.0	8.2	1.8	10.4	14.7
PB4	91.1	7.8	1.1	13.4	0.0
PB5	90.1	8.6	1.3	11.0	0.0
PB6	90.9	7.6	1.5	10.8	16.9
PB7	89.4	8.6	2.0	5.5	8.3
PB8	90.7	7.8	1.5	11.6	0.0

	LR	GNR	oxA_NAc	oxSer	SDEG	MDIS
PB1	6.1	2.5	0.0	8.8	2.33	604
PB2	6.1	2.6	0.0	4.2	2.32	602
PB3	5.8	1.9	0.0	5.6	2.32	602
PB4	6.5	2.2	0.0	0.0	2.31	601
PB5	5.7	1.7	0.0	10.8	2.34	604
PB6	6.1	1.8	0.0	0.0	2.34	604
PB7	5.9	2.0	0.0	2.9	2.34	604
PB8	6.3	3.3	0.0	3.0	2.35	605

Table S5 – Results of the HSQC quantification for the Plackett-Burman design experiments done to evaluate the method robustness

	Hep	DS	ChS
Weight	-0.14	-0.06	0.19
Buffer pH	-0.2	0.06	0.14
Temperature	-0.91	0.65	0.26
Phase	-0.64	0.19	0.45
DM1	0.15	-0.25	0.1
DM2	0.28	0.07	-0.34
DM3	0.08	-0.05	-0.03

	$A_{NH2,6x}$	$A_{NAc,6x}$-(G)	$A_{NAc,6x}$-(I)	$A_{NS,6x}$-(G)	$A_{NS,6x}$-(I)	$A_{NS,6x}$-(I2S)
Weight	-0.11	-0.14	0.22	0.38	0.31	-1.24
Buffer pH	-0.01	0.03	-0.06	-0.45	0.22	0.72
Temperature	-0.06	-0.09	0.07	0.13	0.39	-0.69
Phase	-0.01	0.86	-0.21	-0.6	0.14	0.76
DM1	-0.04	-0.27	0.07	-0.31	0.64	0.4
DM2	-0.03	-0.5	0.17	0.36	-0.07	-0.6
DM3	-0.01	0.7	-0.07	-0.62	0.09	0.11

	$A_{NS,6x}$-(GalA)+Unk	$A_{NS,3S,6x}$	$A_{NAc,6x}$αRed	$A_{NS,6x}$αRed
Weight	0.36	0.07	0.28	0.05
Buffer pH	-0.36	-0.12	0	0.17
Temperature	0.16	-0.03	-0.02	-0.01
Phase	-1.2	0.3	-0.03	-0.18
DM1	-0.68	0.36	0.04	-0.05
DM2	0.68	-0.13	0.12	0.17
DM3	-0.16	-0.21	-0.15	0.16

	G-($A_{NAc,6x}$)	G-($A_{NS,6x}$)	G-($A_{NS,3S,6x}$)
Weight	0.23	0.01	0.33
Buffer pH	0.26	-0.52	0.02
Temperature	-0.19	-0.06	-0.08
Phase	-0.06	0.07	-0.27
DM1	0.12	-0.57	0.31
DM2	0.16	-0.44	-0.19
DM3	-0.3	0.49	0.13

	I-(ANy')	I-(ANy',6S)	I2S-(ANH2,6x)	I2S-(ANy',3x,6x)	2,3-epoxide
Weight	0.58	0.3	0.23	-1.52	-0.17
Buffer pH	0.3	0.11	-0.19	0.2	-0.17
Temperature	-0.26	0.19	-0.22	0.45	0.17
Phase	0.1	-0.2	0.19	0.01	0.17
DM1	-0.08	-0.25	-0.09	0.73	-0.17
DM2	0.09	-0.03	-0.16	0.73	-0.17
DM3	-0.07	-0.28	0.03	-0.17	0.17

	LR	DSulf	% 6S	G_{NR}	$A_{NS,6X}$ from ^{2}A	$A_{NS,3S,6X}$ from ^{2}A	Disacch Mass
Weight	0.13	-0.02	-0.55	0.09	-0.66	0.42	-1.91
Buffer pH	-0.1	0.002	0.29	-0.19	0.22	-0.19	0.21
Temperature	-0.39	0.005	0.17	-0.42	-0.57	0.68	0.47
Phase	0.11	0.007	0.86	0.74	-0.27	-0.35	1.02
DM1	0.05	0.009	-0.34	0.25	0.34	-0.14	0.8
DM2	0.16	0.007	0.06	0.36	-0.18	0.42	0.67
DM3	0.18	-0.002	0.63	-0.21	-0.34	-0.13	0

Table S6 – Plackett-Burman design - effects of factors on the HSQC quantification.

**Condition 1**<br>Operator A<br>Spectr. 500MHz	**Condition 2**<br>Operator A<br>Spectr. 600MHz	**Condition 3**<br>Operator B<br>Spectr. 500MHz	**Condition 4**<br>Operator B<br>Spectr. 600MHz
replicate (1,#1)	*replicate (2,#1)*	*replicate (3,#1)*	*replicate (4,#1)*
replicate (1,#2)	*replicate (2,#2)*	*replicate (3,#2)*	*replicate (4,#2)*
replicate (1,#3)	*replicate (2,#3)*	*replicate (3,#3)*	*replicate (4,#3)*
replicate (1,#4)	*replicate (2,#4)*	*replicate (3,#4)*	*replicate (4,#4)*

Let c be the number of conditions $(c = 4)$, m the number of replicates for each condition $(r = 4)$ and x_{ij} the value obtained in the j-th replicate of i-th condition for fragment x. Define

$$\bar{x}_i = \frac{1}{m} \cdot \sum_{j=1}^{m} x_{ij}$$

$$\bar{\bar{x}} = \frac{1}{cm} \sum_{i=1}^{c} \sum_{j=1}^{m} x_{ij}$$

$$s_1^2 = \frac{m}{c-1} \cdot \sum_{j=1}^{c} (\bar{x}_i - \bar{\bar{x}})^2$$

$$s_2^2 = \frac{1}{c(m-1)} \cdot \sum_{i=1}^{c} \sum_{j=1}^{m} \left(x_{ij} - \bar{x}_i\right)^2$$

The p-value of s_1^2/s_2^2 for the F-distribution with $c-1$ degrees of freedom at the numerator and $m-1$ degrees of freedom at the denominator was computed. Significance of conditions on fragment x evaluation was set at 5%.

Table S7 – Intermediate precision: experiments design (four conditions; four replicates for each condition) and formulae for assess significance of conditions.

	A_NAc6X_G	A_NAc6X_I	A_NS6X_G	A_NS6X_I	A_NS6X_I_2S	A_NS6X_GalA	A_NS6X_Epox	A_NH26X	A_NAc6X	A_NS3S6X	A_NAc6XaRed	A_NS6XaRed	A_NS6X	A_6S
replicate (1,#1)	14.4	1.0	9.0	9.1	58.2	0.0	0.4	2.2	16.6	4.7	1.3	-0.1	76.5	73.2
replicate (1,#2)	13.8	0.9	10.4	9.1	58.2	0.0	0.0	2.2	15.8	4.7	1.2	-0.4	77.3	73.5
replicate (1,#3)	13.6	0.9	9.3	10.5	57.8	0.0	0.0	2.1	15.5	4.7	1.0	0.1	77.7	73.4
replicate (1,#4)	15.1	0.8	9.9	10.4	54.7	0.9	0.0	2.4	16.8	4.9	0.9	-0.1	76.0	73.2
replicate (2,#1)	14.6	0.7	9.4	9.1	58.0	0.0	0.0	2.3	16.4	5.4	1.2	-0.5	75.9	75.2
replicate (2,#2)	14.2	0.8	9.4	9.3	59.3	0.0	0.0	1.3	15.6	4.7	0.6	0.4	78.3	74.7
replicate (2,#3)	14.4	0.8	10.3	9.3	57.8	0.0	0.0	2.1	16.1	4.7	0.9	-0.3	77.2	74.7
replicate (2,#4)	13.7	0.6	9.3	8.2	59.8	0.0	0.0	1.8	15.6	5.2	1.3	0.1	77.3	74.8
replicate (3,#1)	14.9	0.6	9.5	9.3	57.3	0.8	0.4	1.9	16.6	4.1	1.0	0.1	77.4	73.2
replicate (3,#2)	15.0	0.6	10.3	8.6	57.5	0.8	0.0	2.1	16.5	4.5	0.9	-0.2	77.0	73.3
replicate (3,#3)	14.6	0.7	9.7	9.3	56.5	2.3	0.0	2.2	16.2	4.0	0.9	-0.2	77.6	72.8
replicate (3,#4)	14.6	1.0	9.7	8.5	58.1	1.0	0.0	2.0	16.4	4.1	0.8	0.2	77.5	73.3
replicate (4,#1)	14.3	0.8	9.0	9.4	58.9	1.1	0.0	2.0	16.1	3.7	0.9	-0.2	78.3	74.6
replicate (4,#2)	13.5	0.7	9.4	9.2	59.5	0.0	0.0	2.3	15.3	4.6	1.1	-0.3	77.8	74.9
replicate (4,#3)	14.9	0.6	9.7	8.9	58.7	0.0	0.0	2.5	16.1	4.2	0.7	-0.2	77.2	74.3
replicate (4,#4)	14.2	0.9	9.3	9.5	59.2	0.0	0.0	1.5	15.7	4.0	0.7	0.7	78.7	75.4

	G_A_NAc6X	G_A_NS6X	G_A_NS3S6X	I_A_NY	I_A_NY6S	I_2S_A_NH26X	I_2S_A_NY3X6X	G_2OH	G_2S	I_2OH	I_2S	GalA	Epox
replicate (1,#1)	6.7	6.9	3.0	3.6	7.2	2.2	69.8	16.6	0.0	10.9	72.0	0.3	0.4
replicate (1,#2)	6.5	7.4	3.6	3.8	7.8	2.7	68.3	17.4	0.0	11.6	71.0	0.0	0.0
replicate (1,#3)	6.8	7.7	4.0	3.5	7.2	2.7	68.1	18.5	0.0	10.6	70.8	0.0	0.0
replicate (1,#4)	6.6	6.9	3.9	3.6	7.4	2.7	68.9	17.4	0.0	11.0	71.6	0.0	0.0
replicate (2,#1)	6.9	7.4	3.4	3.1	8.0	2.1	69.2	17.6	0.0	11.1	71.3	0.0	0.0
replicate (2,#2)	7.1	7.6	3.4	2.9	8.2	2.0	68.8	18.1	0.0	11.1	70.8	0.0	0.0
replicate (2,#3)	6.7	7.1	3.7	3.3	8.1	2.3	68.8	17.6	0.0	11.4	71.1	0.0	0.0
replicate (2,#4)	7.7	8.3	2.9	3.5	7.4	2.3	67.9	18.9	0.0	11.0	70.2	0.0	0.0
replicate (3,#1)	6.0	7.1	3.3	4.2	6.9	2.2	69.9	16.4	0.0	11.1	72.2	0.0	0.4
replicate (3,#2)	6.6	6.7	3.4	4.1	7.7	1.9	69.6	16.7	0.0	11.8	71.5	0.0	0.0
replicate (3,#3)	6.5	7.2	3.2	3.8	7.5	2.3	69.6	16.8	0.0	11.3	71.9	0.0	0.0
replicate (3,#4)	6.5	7.0	3.0	3.4	7.5	2.1	70.5	16.4	0.0	10.9	72.7	0.0	0.0
replicate (4,#1)	6.8	6.6	3.7	4.3	7.9	2.2	68.5	17.1	0.0	12.2	70.7	0.0	0.0
replicate (4,#2)	6.5	7.2	3.5	4.1	7.7	2.2	68.9	17.2	0.0	11.7	71.1	0.0	0.0
replicate (4,#3)	7.3	8.2	3.2	3.5	7.5	2.0	68.3	18.7	0.0	11.0	70.3	0.0	0.0
replicate (4,#4)	7.0	6.3	3.5	3.4	7.9	2.3	69.6	16.8	0.0	11.3	72.0	0.0	0.0

Table S8.1 – Results of the HSQC quantification for the intermediate precision experiments

	LR	GNR	oxA_NAc	oxSer	SDEG	MDIS
replicate (1,#1)	6.0	2.1	0.0	10.6	2.31	602
replicate (1,#2)	6.0	2.3	0.0	6.0	2.31	601
replicate (1,#3)	6.4	2.2	0.0	4.2	2.31	601
replicate (1,#4)	6.1	2.5	0.0	10.2	2.31	601
replicate (2,#1)	6.0	2.7	0.0	7.9	2.33	604
replicate (2,#2)	5.9	2.0	0.0	10.7	2.33	603
replicate (2,#3)	6.5	2.1	0.0	2.1	2.32	603
replicate (2,#4)	6.1	2.7	0.0	3.6	2.33	603
replicate (3,#1)	5.7	2.2	0.0	2.6	2.31	601
replicate (3,#2)	5.7	1.8	0.0	11.9	2.31	601
replicate (3,#3)	5.9	2.4	0.0	9.0	2.30	601
replicate (3,#4)	6.0	2.3	0.0	7.5	2.32	602
replicate (4,#1)	5.9	2.2	0.0	4.5	2.31	601
replicate (4,#2)	6.1	2.6	0.0	7.7	2.33	603
replicate (4,#3)	5.8	2.3	0.0	7.0	2.30	601
replicate (4,#4)	5.9	1.9	0.0	8.7	2.34	604

	Hep	DS	ChS	DS2S	ChS6S
replicate (1,#1)	87.8	9.7	2.5	13.0	27.0
replicate (1,#2)	88.7	9.1	2.2	15.5	32.9
replicate (1,#3)	89.7	8.4	1.9	12.8	36.3
replicate (1,#4)	89.5	8.4	2.1	12.0	14.2
replicate (2,#1)	90.5	7.5	2.0	11.3	24.6
replicate (2,#2)	89.2	8.5	2.3	13.0	15.0
replicate (2,#3)	90.7	7.6	1.7	11.7	21.7
replicate (2,#4)	90.3	7.9	1.8	14.7	0.0
replicate (3,#1)	90.3	7.9	1.8	8.5	8.9
replicate (3,#2)	89.4	8.7	1.9	13.8	24.5
replicate (3,#3)	90.2	8.4	1.4	15.0	0.0
replicate (3,#4)	90.2	7.8	2.0	12.7	15.0
replicate (4,#1)	91.3	7.3	1.4	15.5	0.0
replicate (4,#2)	89.7	8.3	2.0	11.0	17.8
replicate (4,#3)	90.2	7.9	1.9	11.4	11.1
replicate (4,#4)	90.3	8.1	1.7	13.3	16.9

Table S8.2 – Results of the HSQC quantification for the intermediate precision experiments

	$A_{NH2,6x}$	$A_{NAc,6x}$	$A_{NS,6X}$	$A_{NS,3S,6x}$
Mean	2.06	16.09	77.34	4.5
Pr>F	0.55	0.23	0.22	0.00
CV	14.35	2.76	1.01	10.9

	Hep	DS	ChS
Mean	89.87	8.21	1.92
Pr>F	0.05	0.05	0.13
CV	0.98	7.91	15.47

	$A_{NH2,6x}$	$A_{NAc,6x}$-(G)	$A_{NAc,6x}$-(I)	$A_{NS,6x}$-(G)	$A_{NS,6x}$-(I)	$A_{NS,6x}$-(I2S)	$A_{NS,3S,6x}$	$A_{NAc,6x}\alpha$Red	$A_{NS,6x}\alpha$Red
Mean	2.06	14.36	0.77	9.6	9.25	58.08	4.5	0.96	-0.07
Pr>F	0.55	0.33	0.2	0.59	0.15	0.06	0.00	0.48	0.94
CV	14.35	3.55	16.72	4.58	6.47	2.14	10.9	23.02	-473.57

	G_{2OH}	I_{2OH}	I_{2S}
Mean	17.38	11.24	71.31
Pr>F	0.06	0.34	0.04
CV	4.65	3.67	1.02

	G-($A_{NAc,6x}$)	G-($A_{NS,6x}$)	G-($A_{NS,3S,6x}$)	I-($A_{Ny'}$)	I-($A_{Ny',6S}$)	I2S-($A_{NH2,6x}$)	I2S-($A_{Ny',3x,6x}$)
Mean	6.76	7.22	3.41	3.63	7.61	2.26	69.05
Pr>F	0.03	0.43	0.34	0.06	0.07	0.02	0.04
CV	6.01	7.53	9.26	10.91	4.76	11.53	1.12

	LR	% 6S	G_{NR}	$A_{NS,6X}$ from ^{2}A	$A_{NS,3S,6X}$ from ^{2}A
Mean	6.00	74.03	2.26	76.39	5.46
Pr>F	0.11	0.00	0.71	0.47	0.43
CV	3.57	1.3	11.93	1.05	7.87

Table S9 – Results of the intermediate precision study for the HSQC quantification. Pr>F is the obtained p-value and CV is the computed coefficient of variation.

Group	Sample	ANAc6X-G	ANAc6X-I	ANS6X-G	ANS6X-I	ANS6X-I2S	ANS6X-GalA	ANS6X-Epox	ANH26X	ANAc6X	ANS3S6X	ANAc6XaRed	ANS6XaRed	A_NS6X	A-6S
	G9907	15.7	0.0	9.5	10.1	56.5	1.3	0.0	1.6	17.1	3.9	0.0	0.0	77.4	73.7
	G9908	17.6	0.0	10.1	9.8	56.4	0.0	0.0	1.1	18.4	4.1	0.0	0.0	76.3	70.5
	G9909	15.5	0.0	10.3	9.5	56.9	0.0	0.0	0.0	17.6	4.1	1.2	0.0	77.3	70.4
	G9911	16.4	0.0	9.4	9.7	56.8	0.0	0.0	1.5	17.9	4.2	0.0	0.0	76.4	69.9
	G9912	14.2	0.0	9.6	8.7	59.5	0.0	0.0	1.8	15.8	4.3	0.0	0.0	78.1	72.7
	G9913	15.3	0.0	11.1	8.9	57.3	0.0	0.0	1.8	16.6	4.3	0.0	0.0	77.2	74.4
	G9914	16.3	0.0	10.4	9.3	57.0	0.0	0.0	2.0	17.6	4.0	0.0	0.0	76.5	71.8
	G9915	16.1	0.0	10.8	9.5	55.2	1.6	0.0	1.7	16.7	4.3	0.0	0.0	77.3	73.6
	G9916	14.2	0.0	9.6	10.0	56.5	0.0	0.0	1.9	16.3	5.5	1.3	0.0	76.3	76.9
	G9917	13.9	0.0	9.4	9.2	58.5	1.5	0.0	1.8	15.3	4.5	0.0	0.0	78.3	76.2
	G9918	15.4	0.0	10.2	9.2	58.7	0.0	0.0	2.3	16.2	3.4	0.0	0.0	78.2	74.2
	G9919	14.6	0.0	10.1	9.0	60.6	0.0	0.0	2.9	14.6	3.8	0.0	0.0	78.7	74.3
	G9920	13.8	0.0	9.4	8.8	60.2	1.2	0.0	2.0	14.4	4.4	0.0	0.0	79.2	77.0
	G9921	15.1	0.0	8.8	9.1	59.0	0.0	0.0	2.1	16.5	4.7	0.0	0.0	76.8	74.5
	G9922	14.4	0.0	9.3	9.4	58.6	0.0	0.0	1.8	15.7	4.8	0.0	0.0	77.7	75.8
	G9923	15.6	0.0	9.6	9.6	57.0	0.0	0.0	1.9	16.9	4.9	0.0	0.0	76.3	74.1
	G9924	15.1	1.0	10.8	9.3	57.3	0.0	0.0	1.2	17.0	4.2	0.9	0.0	77.6	70.6
	G9925	13.4	0.0	9.2	9.3	59.7	0.0	0.0	1.8	15.1	5.1	0.0	0.0	78.0	75.7
	G9926	15.1	0.0	10.0	8.5	58.0	0.0	0.0	2.1	16.2	4.8	0.0	0.0	77.0	73.6
	G9927	17.6	0.0	9.9	9.1	55.7	0.0	0.0	1.1	19.2	4.1	0.0	0.0	75.6	69.1
	G9928	16.9	0.0	10.2	9.5	55.7	0.0	0.0	1.8	18.6	3.8	0.0	0.0	75.8	68.3
	G9929	16.0	0.0	10.1	10.5	56.6	0.0	0.0	1.9	17.7	3.7	1.2	0.0	76.8	71.0
	G9930	15.4	0.0	9.0	10.5	56.1	0.0	0.0	1.9	17.0	5.3	0.0	0.0	75.8	73.1
	G9932	14.7	0.0	9.8	10.2	56.5	1.9	0.0	1.7	16.1	3.5	0.0	0.0	78.8	75.2
	G9933	17.6	0.0	10.7	8.8	55.9	0.0	0.0	1.4	18.6	4.3	0.0	0.0	75.7	68.5
	G9934	12.2	0.9	10.0	9.3	59.2	1.3	0.0	1.7	14.1	4.3	1.1	0.0	79.8	75.3
	G9935	13.9	0.0	10.0	9.3	58.2	1.0	0.0	1.9	15.3	4.2	0.9	0.0	78.7	73.5
	G9936	15.5	0.0	9.2	10.9	56.3	0.0	0.0	2.0	16.6	4.3	0.0	0.0	77.1	73.2
C	G9937	18.8	0.0	10.7	9.8	51.6	2.2	0.0	1.3	20.4	3.1	1.0	0.0	75.2	67.6
	G9938	15.1	1.1	10.3	9.8	57.1	0.0	0.0	1.9	17.0	4.0	0.0	0.0	77.2	71.3
	G9939	15.1	1.0	10.1	8.9	58.4	0.0	0.0	2.0	16.9	3.6	0.0	0.0	77.5	71.8
	G9940	13.8	0.0	9.3	8.6	60.6	0.0	0.0	1.6	15.3	4.2	0.0	0.0	78.8	74.8
	G9941	14.6	0.0	9.7	8.8	59.1	0.0	0.0	1.6	16.3	4.0	0.0	0.0	78.0	72.9
	G9942	15.1	0.0	10.2	8.5	58.3	0.0	0.0	1.8	16.7	4.5	0.0	0.0	77.0	71.7
	G9943	14.1	0.0	9.8	10.4	57.6	0.0	0.0	2.1	16.2	3.9	1.5	0.0	77.9	69.3
	G9944	11.7	0.0	9.0	9.8	62.0	0.0	0.0	2.2	13.1	4.2	0.0	0.0	80.6	77.6
	G9945	12.5	0.0	9.4	9.0	59.8	1.1	0.0	2.1	14.1	3.9	0.0	0.0	79.9	73.7
	G9946	14.7	0.0	10.0	9.3	57.2	0.0	0.0	1.9	16.8	4.4	0.0	0.0	76.9	74.8
	G9947	14.1	0.0	10.2	9.6	58.3	0.0	0.0	2.2	15.6	3.8	0.0	0.0	78.5	72.4
C	G9948	19.0	0.0	10.2	8.1	56.8	0.0	0.0	1.8	20.0	3.2	0.0	0.0	75.1	68.5
	G9949	12.9	0.0	9.4	8.4	62.6	0.0	0.0	2.6	13.4	4.0	0.0	0.0	80.0	74.7
	G9950	14.8	0.0	9.7	9.6	58.1	0.0	0.0	2.0	16.0	4.8	0.0	0.0	77.3	74.3
B	G12131	11.9	0.0	9.7	9.0	61.6	0.0	0.0	2.3	12.6	4.9	0.0	0.0	80.2	77.7
	G12132	15.0	0.0	10.1	8.5	56.5	1.1	0.0	1.9	16.4	5.3	0.0	0.0	76.4	74.6
B	G12133	11.1	0.0	11.2	9.9	60.4	0.0	0.0	2.8	11.6	4.9	0.0	0.0	80.7	77.6
	G12134	14.9	0.0	10.0	9.7	57.0	0.0	0.0	1.8	16.4	4.8	0.9	0.0	77.0	76.3
B	G12135	12.3	0.0	9.7	7.8	61.4	0.0	0.0	2.4	13.2	5.3	0.0	0.0	79.1	79.0
	G12136	14.0	0.0	8.4	9.2	60.5	0.0	0.0	2.3	16.1	3.7	1.4	0.0	77.8	72.8
	G12137	14.2	0.0	9.5	9.5	55.9	1.7	0.0	1.9	15.8	5.3	0.0	0.0	77.0	74.1
	G12138	12.0	0.0	8.7	9.1	60.0	0.0	0.0	3.0	13.9	5.9	1.0	0.0	77.2	77.0
	G12139	16.1	0.0	9.2	8.8	56.6	1.7	0.0	1.5	17.7	4.4	0.0	0.0	76.4	70.9
B	G12141	13.2	0.0	8.9	8.1	62.8	0.0	0.0	2.1	13.8	4.1	0.0	0.0	80.0	79.8
B	G12142	13.5	0.0	10.2	8.3	59.5	0.0	0.0	2.6	14.5	5.1	0.0	0.0	77.8	78.0
	G12147	20.0	0.0	10.2	9.1	53.7	0.0	0.0	1.8	21.1	4.1	0.0	0.0	72.9	66.5
	G12149	14.8	0.0	10.7	8.6	57.2	0.0	0.0	2.0	16.5	4.5	0.0	0.0	77.0	75.0
	G12150	13.9	0.0	9.7	9.7	57.6	0.0	0.0	2.2	15.3	4.5	0.0	0.0	77.9	72.9
	G12151	18.3	0.0	9.2	10.6	53.1	0.0	0.0	1.3	20.1	4.6	0.0	0.0	74.1	67.2
C	G12152	17.5	0.0	10.3	8.8	57.6	0.0	0.0	0.0	18.4	3.7	0.0	0.0	76.8	68.4
C	G12153	18.5	0.0	8.9	9.6	56.1	0.0	0.0	1.8	19.4	4.1	0.0	0.0	74.7	67.3
C	G12154	18.6	0.0	11.0	7.6	57.0	0.0	0.0	0.0	19.3	3.9	0.0	0.0	76.8	69.4
	G12155	16.8	0.0	9.2	9.6	55.8	0.0	0.0	2.2	18.7	4.6	0.0	0.0	74.5	69.9
	G12156	19.9	0.0	10.7	9.3	55.2	0.0	0.0	0.0	19.9	3.9	0.0	0.0	76.1	63.3
	G12158	14.8	0.0	9.8	9.5	56.8	0.0	0.0	1.5	16.5	5.5	0.0	0.0	76.5	73.2
	G12161	11.8	0.0	10.0	8.5	60.7	0.0	0.0	2.6	12.2	5.9	0.0	0.0	79.3	79.2
B	G12163	15.0	1.0	9.8	8.7	56.6	0.0	0.0	2.0	16.9	5.5	0.0	0.0	75.5	74.7
	G12164	14.1	0.0	9.1	8.8	59.6	0.0	0.0	1.9	15.8	4.5	0.0	0.0	77.8	73.9
A	G12166	14.75	0	9.07	8.47	60.12	0	0	2.04	14.75	5.4	0	0	77.81	75.97
	G12169	13.4	0.0	9.7	8.8	57.9	0.0	0.0	2.0	15.4	5.2	0.0	0.0	77.4	73.7
A	G12170	14.47	0	10.56	7.97	59.82	0.96	0	0	14.47	5.13	0	1.09	80.4	75.92
	G12174	13.7	0.0	8.8	9.9	58.3	0.0	0.0	2.4	14.9	5.9	0.0	0.0	76.8	73.2
	G12175	14.8	0.0	9.5	8.6	57.9	0.0	0.0	2.2	16.6	5.2	0.0	0.0	76.0	73.1
	G12177	16.1	0.0	9.1	8.1	58.5	0.0	0.0	2.2	17.8	4.5	0.0	0.0	75.5	71.1
A	G12179	12.6	0.0	10.5	9.6	58.8	2.1	0.0	0.0	13.7	5.2	0.0	0.0	81.1	77.0
A	G12180	12.72	0.89	10.25	8.64	59.59	0.97	0	0.99	13.62	5.03	0	0.92	80.37	78.9
	G12181	15.42	0	9.59	9.22	58.41	0	0	1.45	15.42	5.4	0	0	77.73	77.74
	average	15.0	0.1	9.8	9.2	57.9	0.3	0.0	1.8	16.3	4.5	0.2	0.0	77.4	73.3
	median	14.8	0.0	9.8	9.2	57.9	0.0	0.0	1.9	16.3	4.4	0.0	0.0	77.3	73.7
	st.dev.	1.94	0.27	0.61	0.67	2.09	0.61	0.00	0.65	1.99	0.66	0.41	0.16	1.64	3.36
	min	11.1	0.0	8.4	7.6	51.6	0.0	0.0	0.0	11.6	3.1	0.0	0.0	72.9	63.3
	max	20.0	1.1	11.2	10.9	62.8	2.2	0.0	3.0	21.1	5.9	1.5	1.1	81.1	79.8

Table S10 - Molar percentages of glucosamine residues of 75 porcine crude heparin samples. NB: 0 value means <LOD

Group	Sample	G_ANAc6X	G_ANS6X	G_ANS3S6X	I_ANY	I_ANY6S	I2S_ANH26X	I2S_ANY3X6X	G2OH	G2S	I2OH	I2S
	G9907	7.5	6.8	3.6	3.5	8.1	2.8	67.7	17.9	0.0	11.7	70.4
	G9908	10.5	6.2	3.6	3.5	6.8	1.4	68.0	20.2	0.0	10.4	69.4
	G9909	9.7	6.7	2.8	3.2	7.8	1.8	67.9	19.2	0.0	11.0	69.8
	G9911	11.0	7.1	2.8	3.2	6.9	1.5	67.5	20.9	0.0	10.1	69.1
	G9912	6.8	7.2	3.5	3.5	7.9	2.2	68.9	17.6	0.0	11.3	71.1
	G9913	6.5	7.3	3.8	4.1	7.1	1.8	69.4	17.6	0.0	11.2	71.2
	G9914	7.8	6.6	3.7	3.4	7.8	2.7	68.1	18.1	0.0	11.1	70.8
	G9915	6.9	7.5	3.0	3.3	6.9	2.3	70.1	17.4	0.0	10.2	72.4
	G9916	6.3	7.4	4.2	2.8	7.9	2.4	69.0	17.9	0.0	10.7	71.4
	G9917	5.4	6.9	3.9	3.4	7.8	2.2	70.4	16.1	0.0	11.2	72.7
	G9918	6.5	8.0	4.1	3.6	6.2	2.8	68.8	18.6	0.0	9.8	71.6
	G9919	6.5	7.1	4.2	2.0	7.9	2.4	70.0	17.8	0.0	9.9	72.4
	G9920	6.6	6.5	3.0	3.2	7.5	2.1	71.2	16.1	0.0	10.6	73.3
	G9921	6.4	6.7	3.0	3.1	7.5	2.1	71.1	16.2	0.0	10.6	73.2
	G9922	6.1	6.9	3.7	3.8	7.5	1.9	70.2	16.7	0.0	11.2	72.1
	G9923	7.4	6.8	4.1	3.5	6.4	2.4	69.5	18.2	0.0	9.9	72.0
	G9924	9.3	7.4	3.8	3.3	6.5	1.7	68.0	20.5	0.0	9.7	69.8
	G9925	6.2	6.5	4.9	3.3	7.4	2.3	69.4	17.6	0.0	10.8	71.7
	G9926	6.9	6.0	4.1	3.8	7.4	2.2	69.7	17.0	0.0	11.2	71.9
	G9927	9.6	7.9	3.3	3.3	7.1	2.1	66.7	20.7	0.0	10.5	68.8
	G9928	9.2	8.4	2.9	3.8	7.6	2.1	65.9	20.6	0.0	11.4	68.1
	G9929	8.4	7.8	3.2	3.4	7.4	1.9	67.8	19.4	0.0	10.8	69.7
	G9930	6.5	7.7	3.9	3.5	8.9	2.7	66.9	18.0	0.0	12.4	69.6
	G9932	6.5	6.9	3.7	3.4	7.9	2.4	69.2	17.1	0.0	11.3	71.6
	G9933	8.8	7.7	3.6	3.2	6.5	2.1	68.1	20.1	0.0	9.6	70.3
	G9934	6.1	7.7	2.8	3.2	8.0	1.4	70.8	16.6	0.0	11.2	72.2
	G9935	7.8	7.1	2.3	3.3	7.9	1.6	70.0	17.2	0.0	11.2	71.6
	G9936	8.2	6.9	3.1	3.4	7.4	2.4	68.6	18.2	0.0	10.9	71.0
C	G9937	10.9	7.3	2.9	3.3	7.7	1.7	65.5	21.1	0.0	10.9	67.2
	G9938	8.0	6.9	3.9	3.6	7.2	2.1	68.4	18.8	0.0	10.8	70.4
	G9939	7.9	6.3	3.4	3.9	6.6	2.5	69.4	17.6	0.0	10.5	71.9
	G9940	6.6	7.4	3.8	3.2	7.7	2.6	68.7	17.8	0.0	10.9	71.3
	G9941	7.1	7.0	3.4	3.2	8.2	2.2	68.9	17.5	0.0	11.4	71.1
	G9942	6.8	7.9	3.8	3.9	6.8	2.1	68.8	18.5	0.0	10.7	70.9
	G9943	8.6	7.8	3.5	4.1	7.6	2.3	66.1	19.9	0.0	11.7	68.4
	G9944	4.4	7.5	3.2	2.3	6.8	2.7	73.2	15.0	0.0	9.1	75.9
	G9945	6.4	7.2	2.7	3.5	7.3	2.7	70.2	16.3	0.0	10.8	72.9
	G9946	6.4	7.6	3.2	3.6	7.5	2.0	69.8	17.2	0.0	11.0	71.8
	G9947	7.5	8.6	2.8	3.0	8.2	2.2	67.6	19.0	0.0	11.2	69.8
C	G9948	8.3	8.0	3.3	3.4	7.2	2.4	67.4	19.6	0.0	10.6	69.8
	G9949	7.3	6.3	2.4	3.4	6.8	2.0	71.8	16.0	0.0	10.2	73.8
	G9950	5.4	7.1	3.4	3.2	7.3	2.7	70.9	15.9	0.0	10.5	73.6
B	G12131	5.5	6.6	4.1	2.4	5.8	3.2	72.3	16.2	0.0	8.2	75.5
	G12132	7.1	6.8	4.0	3.1	7.0	2.3	69.7	17.9	0.0	10.2	72.0
B	G12133	4.7	6.5	4.0	2.6	6.7	3.9	71.7	15.1	0.0	9.3	75.6
	G12134	7.4	6.1	4.0	2.5	5.8	2.6	71.5	17.6	0.0	8.3	74.1
B	G12135	4.6	7.1	3.1	2.6	6.8	3.0	72.7	14.9	0.0	9.4	75.7
	G12136	5.9	7.6	3.4	3.1	6.5	2.8	70.7	16.9	0.0	9.6	73.5
	G12137	7.4	6.9	4.1	3.6	7.9	2.4	67.8	18.4	0.0	11.5	70.1
	G12138	5.5	7.3	4.1	3.2	7.1	3.0	69.8	16.9	0.0	10.3	72.8
	G12139	9.0	6.8	3.1	3.0	7.2	1.8	69.1	18.9	0.0	10.2	70.9
B	G12141	4.2	8.0	2.6	2.4	6.0	3.1	73.7	14.8	0.0	8.4	76.8
B	G12142	4.5	6.4	3.9	2.1	6.6	2.9	73.6	14.8	0.0	8.7	76.5
	G12147	10.5	7.9	3.5	4.0	8.0	1.9	64.2	21.9	0.0	12.0	66.0
	G12149	6.4	6.9	2.6	3.5	7.0	2.3	71.3	15.8	0.0	10.6	73.6
	G12150	7.8	6.6	3.7	3.1	7.3	2.3	69.1	18.1	0.0	10.4	71.4
	G12151	11.1	6.6	3.0	3.0	8.1	1.8	66.4	20.7	0.0	11.1	68.2
C	G12152	10.0	7.3	4.5	3.4	7.0	1.6	66.2	21.8	0.0	10.4	67.8
C	G12153	7.8	8.2	2.9	3.2	8.3	0.0	68.7	18.9	0.0	11.5	69.6
C	G12154	9.8	6.5	5.8	3.8	6.9	1.8	65.6	22.0	0.0	10.7	67.3
	G12155	8.6	7.4	4.5	3.5	7.6	2.0	66.5	20.4	0.0	11.1	68.5
	G12156	11.4	6.9	4.1	4.5	7.7	2.2	63.2	22.4	0.0	12.2	65.4
	G12158	7.6	7.5	4.0	2.2	7.8	1.9	69.0	19.1	0.0	10.0	70.9
	G12161	5.1	6.6	4.0	2.3	6.3	2.9	72.9	15.6	0.0	8.5	75.8
B	G12163	8.1	6.5	4.0	3.0	7.6	2.7	68.2	18.5	0.0	10.6	70.9
	G12164	6.2	6.7	3.6	3.2	7.1	1.6	71.6	16.5	0.0	10.3	73.2
A	G12166	6.28	7.41	3.02	2.86	6.34	1.74	72.35	16.71	0	9.2	74.08
	G12169	6.9	6.9	4.8	3.8	7.4	2.3	67.8	18.6	0.0	11.3	70.1
A	G12170	6.61	5.76	2.61	2.76	5.73	ND	75.77	14.99	0.75	8.49	75.77
	G12174	7.3	7.5	3.4	3.1	7.2	1.9	69.6	18.2	0.0	10.3	71.5
	G12175	8.2	7.1	4.0	3.0	7.5	2.5	67.8	19.2	0.0	10.5	70.3
	G12177	7.3	8.8	3.4	3.3	7.6	2.2	67.4	19.5	0.0	10.9	69.6
A	G12179	6.6	6.6	2.9	2.2	7.1	0.0	72.9	16.1	0.0	9.4	73.5
A	G12180	5.46	6.04	2.53	2.64	6.55	1.46	75.31	14.04	0	9.19	76.78
	G12181	6.36	6.41	3.72	3.01	7.35	1.76	71.39	16.49	0	10.36	73.15
	average	7.3	7.1	3.5	3.2	7.2	2.2	69.4	18.0	0.0	10.5	71.5
	median	7.1	7.1	3.6	3.3	7.3	2.2	69.1	17.9	0.0	10.6	71.4
	st.dev.	1.67	0.63	0.63	0.50	0.64	0.59	2.40	1.92	0.09	0.92	2.49
	min	4.2	5.8	2.3	2.0	5.7	0.0	63.2	14.0	0.0	8.2	65.4
	max	11.4	8.8	5.8	4.5	8.9	3.9	75.8	22.4	0.8	12.4	76.8

Table S11 - Molar percentages of uronic acids residues of 75 porcine crude heparin samples.

NB: 0 value means <LOD

Group	Sample	Hep	DS	ChS	DS2S*	ChS6S*
	G9907	94.3	4.6	1.0	0.0	0.0
	G9908	86.9	6.5	6.6	0.0	34.7
	G9909	85.8	7.1	7.1	0.0	40.6
	G9911	86.6	5.9	7.5	0.0	38.2
	G9912	89.9	7.8	2.3	12.4	28.8
	G9913	90.2	7.6	2.2	10.2	23.3
	G9914	88.2	9.2	2.6	10.5	27.1
	G9915	91.3	6.7	1.9	0.0	0.0
	G9916	89.3	8.3	2.4	0.0	25.8
	G9917	87.3	10.5	2.1	9.2	17.0
	G9918	92.7	6.6	0.0	0.0	0.0
	G9919	95.0	4.3	0.0	0.0	0.0
	G9920	89.3	8.9	1.9	10.9	34.4
	G9921	87.7	9.6	2.7	10.7	28.4
	G9922	88.1	10.0	1.9	11.1	40.3
	G9923	83.6	13.5	2.9	9.5	33.8
	G9924	88.6	6.2	5.2	0.0	39.1
	G9925	87.7	11.0	1.2	9.0	0.0
	G9926	90.2	9.0	0.0	8.1	0.0
	G9927	90.4	7.0	2.5	10.8	26.7
	G9928	91.3	6.0	2.7	0.0	41.8
	G9929	90.4	7.0	2.7	8.7	23.7
	G9930	89.2	8.7	2.1	0.0	0.0
	G9932	92.7	6.1	1.3	0.0	0.0
	G9933	89.2	7.6	3.2	0.0	33.3
	G9934	98.1	1.4	0.0	0.0	0.0
	G9935	98.3	1.7	0.0	0.0	0.0
	G9936	87.8	8.4	3.7	7.5	16.0
C	G9937	86.5	11.2	2.2	8.8	20.1
	G9938	89.6	7.8	2.6	13.1	30.3
	G9939	91.0	6.9	2.1	12.7	31.3
	G9940	87.9	9.7	2.4	11.7	22.0
	G9941	90.0	8.9	1.2	0.0	0.0
	G9942	91.0	7.3	1.6	7.6	13.4
	G9943	83.6	13.0	3.5	10.8	26.3
	G9944	88.9	10.5	0.0	9.1	0.0
	G9945	90.5	8.9	0.0	8.3	0.0
	G9946	88.8	8.7	2.5	11.9	25.6
	G9947	93.3	5.6	1.1	0.0	0.0
C	G9948	82.6	9.8	7.7	10.8	35.9
	G9949	91.1	7.4	1.5	0.0	0.0
	G9950	87.8	10.4	1.7	12.8	34.0
B	G12131	84.6	13.5	1.8	8.8	18.2
	G12132	89.5	8.5	2.0	13.1	18.7
B	G12133	84.7	13.8	1.5	9.5	0.0
	G12134	89.8	8.7	1.5	6.6	0.0
B	G12135	88.0	11.4	0.0	12.9	0.0
	G12136	88.2	10.2	1.6	5.6	19.8
	G12137	91.3	6.1	2.6	0.0	30.9
	G12138	91.7	7.9	0.0	11.6	0.0
	G12139	94.4	4.7	0.9	0.0	43.6
B	G12141	83.5	14.9	1.7	11.3	0.0
B	G12142	81.8	16.6	1.6	12.5	0.0
	G12147	85.2	8.7	6.0	6.1	41.7
	G12149	90.8	7.2	2.0	8.2	26.3
	G12150	90.5	8.1	1.4	10.9	0.0
	G12151	91.9	5.0	3.0	0.0	33.1
C	G12152	79.9	13.5	6.6	7.1	46.4
C	G12153	83.7	10.3	6.0	0.0	32.1
C	G12154	86.1	7.8	6.2	0.0	24.6
	G12155	89.4	8.3	2.3	0.0	0.0
	G12156	85.0	8.7	6.3	0.0	39.6
	G12158	87.2	9.6	3.2	9.7	37.5
	G12161	86.7	12.0	1.3	12.3	0.0
B	G12163	89.1	9.3	1.6	12.8	42.7
	G12164	89.8	9.4	0.0	0.0	0.0
A	G12166	98.44	1.56	0	0	0
	G12169	87.7	9.9	2.4	12.1	32.8
A	G12170	99.17	0	0	0	0
	G12174	87.9	10.0	2.2	7.5	49.7
	G12175	88.4	9.3	2.3	14.6	33.7
	G12177	87.2	9.7	3.2	11.5	23.6
A	G12179	100.0	0.0	0.0	0.0	0.0
A	G12180	98.67	0	0	0	0
	G12181	82.43	16.52	1.05	10.15	0
	average	89.3	8.3	2.3	6.2	18.5
	median	89.2	8.7	2.0	8.1	22.0
	st.dev.	4.10	3.38	1.95	5.32	16.62
	min	79.9	0.0	0.0	0.0	0.0
	max	100.0	16.6	7.7	14.6	49.7

Table S12 – GAG compositions of 75 porcine crude heparin samples. DS2S* and ChS6S* indicate the percentage of IdoA2S and ChS6S (chondroitin C) of dermatan and chondroitin components, respectively. NB: 0 value means <LOD

Group	Sample	LR	GNR	oxANAc	oxSer	SDEG
	G9907	5.0	2.4	0.0	0.0	2.29
	G9908	5.1	2.2	0.0	0.0	2.24
	G9909	5.4	3.1	0.0	0.0	2.26
	G9911	4.7	2.4	0.0	0.0	2.24
	G9912	6.6	2.3	0.0	0.0	2.30
	G9913	6.5	2.5	0.0	3.1	2.31
	G9914	6.8	2.7	0.0	5.2	2.27
	G9915	5.9	2.5	0.0	0.0	2.32
	G9916	5.2	2.6	0.0	0.0	2.36
	G9917	5.6	2.7	0.0	9.6	2.36
	G9918	6.2	2.4	0.0	0.0	2.31
	G9919	6.1	2.5	0.0	0.0	2.33
	G9920	6.3	2.8	0.0	5.7	2.38
	G9921	6.7	2.3	0.0	7.2	2.34
	G9922	6.2	2.5	0.0	0.0	2.35
	G9923	6.0	2.6	0.0	0.0	2.32
	G9924	5.1	2.6	0.0	0.0	2.26
	G9925	5.5	2.4	0.0	8.2	2.36
	G9926	6.2	2.4	0.0	0.0	2.32
	G9927	6.3	2.6	0.0	0.0	2.22
	G9928	6.3	2.7	0.0	13.5	2.20
	G9929	5.4	2.2	0.0	0.0	2.25
	G9930	5.4	2.1	0.0	0.0	2.29
	G9932	5.6	2.6	0.0	0.0	2.33
	G9933	5.5	2.9	0.0	0.0	2.23
	G9934	4.3	2.3	0.0	7.9	2.36
	G9935	5.1	2.1	0.0	10.0	2.32
	G9936	5.8	2.6	0.0	0.0	2.30
C	G9937	4.1	2.8	0.0	17.7	2.16
	G9938	6.8	2.6	0.0	8.0	2.27
	G9939	6.6	2.5	0.0	4.5	2.28
	G9940	6.0	2.0	0.0	6.3	2.33
	G9941	6.4	2.5	0.0	6.3	2.30
	G9942	6.6	2.6	0.0	5.0	2.29
	G9943	6.2	3.7	0.0	8.2	2.23
	G9944	5.5	2.3	0.0	0.0	2.42
	G9945	5.7	2.6	0.0	0.0	2.34
	G9946	5.8	2.2	0.0	12.5	2.32
	G9947	5.5	2.4	0.0	0.0	2.28
C	G9948	7.1	2.7	0.0	12.5	2.20
	G9949	5.2	2.6	0.0	0.0	2.37
	G9950	6.5	2.8	0.0	4.0	2.35
B	G12131	6.1	2.1	0.0	3.9	2.43
	G12132	5.7	2.1	0.0	0.0	2.34
B	G12133	5.5	2.0	0.0	0.0	2.44
	G12134	5.8	2.1	0.0	0.0	2.37
B	G12135	5.9	1.8	0.0	0.0	2.44
	G12136	6.2	1.9	0.0	0.0	2.32
	G12137	6.5	2.3	0.0	0.0	2.32
	G12138	6.0	2.6	0.0	0.0	2.39
	G12139	5.9	2.0	0.0	0.0	2.27
B	G12141	5.6	2.2	0.0	0.0	2.45
B	G12142	5.7	2.1	0.0	0.0	2.43
	G12147	6.2	2.0	0.0	5.3	2.14
	G12149	5.7	2.3	0.0	0.0	2.35
	G12150	6.4	2.3	0.0	0.0	2.31
	G12151	5.2	3.0	0.0	8.4	2.19
C	G12152	6.1	2.2	0.0	0.0	2.20
C	G12153	5.6	0.0	0.0	0.0	2.20
C	G12154	6.2	0.0	0.0	0.0	2.21
	G12155	6.0	2.4	0.0	0.0	2.22
	G12156	6.2	0.0	0.0	0.0	2.13
	G12158	6.4	1.9	0.0	11.4	2.32
	G12161	5.4	2.0	0.0	12.5	2.46
B	G12163	6.4	2.5	0.0	0.0	2.32
	G12164	6.4	2.9	0.0	0.0	2.34
A	G12166	4.69	2.17	ND	100	2.39
	G12169	6.4	2.6	0.0	10.6	2.32
A	G12170	5.63	2.76	ND	100	2.43
	G12174	6.3	1.9	0.0	6.8	2.33
	G12175	6.2	2.1	0.0	0.0	2.30
	G12177	7.0	2.4	0.0	7.8	2.25
A	G12179	4.4	1.7	0.0	100.0	2.42
A	G12180	4.36	2.02	ND	100	2.46
	G12181	6.39	2.02	ND	0	2.39
	average	5.9	2.3	0.0	8.3	2.3
	median	6.0	2.4	0.0	0.0	2.3
	st.dev.	0.65	0.58	0.00	22.35	0.08
	min	4.1	0.0	0.0	0.0	2.1
	max	7.1	3.7	0.0	100.0	2.5

Table S13 - Linkage region and sulfation degree of 75 porcine crude heparin samples. NB: 0 value means <LOD

The alignment algorithm was developed by a modification of the algorithm contained in the R “ptw” (Parametric Time Warping) alignment package[1] and works as follows: if the original abscissa is 1, …, n, then the signal of the aligned spectrum at k will be equal to the intensity of the original spectrum at

$$s(k) = k + \sum_{i=1}^{d} a_i \cdot sin\left(\pi \frac{i(k-1)}{n-1}\right)$$

The user has to select d and the initial values for the a_i coefficients, which are optimized by the software by minimizing the distance between the aligned spectrum and the reference spectrum.
If $s(k)$ is not an integer, the algorithm interpolates between the intensities at the two integers closest to $s(k)$.
The equally spaced abscissa values (ppm) of the NMR spectrum are mapped to and from 1, ..., n in the obvious way before and after the application of the algorithm.

[1] T.G. Bloemberg, J. Gerretzen, H.J.P. Wouters, J. Gloerich, M. van Dael, H.J.C.T. Wessels, L.P. van den Heuvel, P.H.C. Eilers, L.M.C. Buydens, R. Wehrens, Improved parametric time warping for proteomics, Chemometr. Intell. Lab. 2010, 104, 65-74. DOI: 10.1016/j.chemolab.2010.04.008. R. Wehrens, T.G. Bloemberg, P.H.C. Eilers, Fast parametric time warping of peak lists, Bioiformatics 2015, 31, 3063-3065. DOI: 10.1093/bioinformatics/btv299.

Table S14 – Alignment algorithm

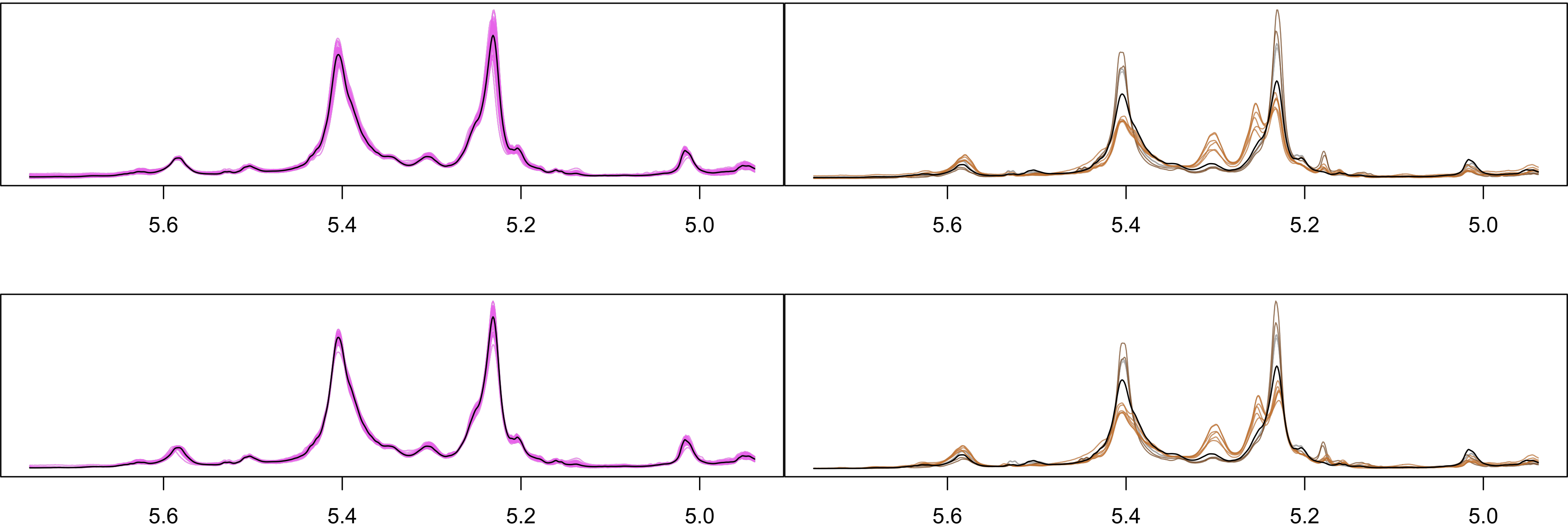

Figure S9 – Alignment algorithm. Black lines=reference spectrum. Pink lines=PMHC spectra. Grey lines=OMHC spectra. Dark brown lines=BMHC spectra. Light brown lines=BLCH spectra. Top panels shows spectra before alignment. Bottom panels show spectra after alignment.
